# Multi-attribute monitoring (MAM) methodology for glycosylated subunit vaccines

**DOI:** 10.1038/s41598-025-24922-8

**Published:** 2025-11-21

**Authors:** Asif Shajahan, Lisa M. Jenkins, Nathan Barefoot, Darielys Maldonado, Jeremy J. Wolff, Yanhong Yang, Lisa A. Kueltzo, Valerie Ficca, Elizabeth Scheideman, Ivan Loukinov, Carl Carruthers, Dorra Benmohamed, Daniel B. Gowetski, Rong Jiang, Sylvie R. Yang, Kevin Carlton, Jason G. Gall, Q. Paula Lei

**Affiliations:** 1 Vaccine Production Program Laboratory, Vaccine Research Center, National Institute of Allergy and Infectious Diseases, National Institutes of Health, Gaithersburg, MD USA; 2 Laboratory of Cell Biology, Center for Cancer Research, National Cancer Institute, National Institutes of Health, Bethesda, MD USA

**Keywords:** Biochemistry, Biological techniques, Biotechnology, Computational biology and bioinformatics, Immunology

## Abstract

Many protein-based vaccines comprise viral surface proteins which are chosen for their ability to stimulate the immune system. These vaccine molecules are often heavily glycosylated, and glycosylation plays critical roles in the immunological and stability properties of vaccines. The structural characterization and product quality attribute monitoring of such complex vaccine therapeutics during process development and manufacturing is very challenging. High throughput monitoring of multiple molecular attributes, particularly glycosylation, of recombinant glycoprotein subunit vaccines are needed to support entire vaccine production processes. Multi-attribute monitoring (MAM) technology involves assessing multiple critical molecular attributes of molecules in one set of analyses in an automated fashion, for product quality attribute requirements. MAM is still in the early development stages and is currently applied to therapeutics with very low levels of glycosylation such as monoclonal antibodies. MAM on glycoproteins with a higher number of glycosylation sites with high glycan heterogeneity such as subunit vaccine molecules is challenging as each glycan site and glycan modification exponentially increases data processing complexity. We developed a MAM workflow to perform detailed structural characterization of subunit protein vaccines, monitoring critical parameters such as intact mass, sequence identity, protein clipping, glycosylation, other post-translational modifications, and host cell proteins (HCP). By using a combination of software tools and product process monitoring strategy, we performed data processing at multiple steps and identified key attributes for each vaccine candidate under the development pipeline. Further, a high-throughput critical attribute monitoring MAM workflow was developed to support the influenza and HIV vaccine development processes including cell line selection, cell clone selection, cell culture optimization, stability study evaluation and final vaccine product characterization.

**Supplementary Information:**

The online version contains supplementary material available at 10.1038/s41598-025-24922-8.

## Introduction

The expression of subunit vaccines from mammalian cell lines yields proteins with post-translational modifications (PTMs)^1,2^. Glycosylation, a common PTM, affects protein folding, stability, virus attachment, and antibody neutralization^3^. Most subunit protein vaccines are heavily glycosylated, influencing their effectiveness and pharmacokinetics^3,4^. In-depth characterization of glycosylation is therefore critical in vaccine development, impacting all stages from cell line selection to stability studies^5^.

PTMs other than glycosylation can also affect the structure and function of the vaccine and need to be characterized^6–8^. The Multi-Attribute Monitoring (MAM) method is a peptide mapping-based method that uses high-resolution mass spectrometry (MS) to improve process control for biopharmaceutical manufacturing^9–12^. (MAM) has emerged as an impactful analytical approach in biopharmaceutical development, particularly for monoclonal antibodies and other relatively simple biologics. Early applications of MAM focused on monitoring attributes such as glycosylation, oxidation, and deamidation using high-resolution mass spectrometry coupled with automated data processing tools. MAM has been implemented in multiple product development pipelines in monitoring glycan compositions, clipping sites, and post-translational modifications under stress and stability conditions. Recent methodological advances in MAM such as optimized digestion protocols, automated sample preparation, and isotope normalization strategies have improved the accuracy of MAM workflows. These advances led to the implementation of MAM under GMP conditions, enabling its use from early development through QC (Quality Control) release testing^13–15^. MAM aims to address the challenges of characterizing large molecule drugs, such as monoclonal antibodies and antibody–drug conjugates to ensure the safety and efficacy of a batch before release^9,16^.

MAM can significantly enhance the characterization of glycosylation profiles in subunit vaccines compared to traditional analytical techniques by offering a more integrated, high-resolution, and site-specific approach. Traditional methods such as HPLC, CE, and ELISA typically assess glycosylation indirectly or in isolation, often requiring multiple assays to evaluate different attributes. In contrast, MAM utilizes high-resolution mass spectrometry to simultaneously monitor multiple critical quality attributes—including glycosylation—within a single analytical workflow. This enables direct identification and quantification of glycan structures at specific sites, detection of glycan heterogeneity, and assessment of site occupancy with greater precision. Additionally, MAM allows for the detection of unexpected modifications and provides deeper insights into glycosylation patterns under varying process conditions. The ability to integrate glycosylation data with other attributes such as intact mass, sequence variants, and post-translational modifications makes MAM a powerful tool for comprehensive structural characterization and process monitoring of complex vaccine candidates^13–15^.

MAM is currently exclusively applied to therapeutics with very low levels of glycosylation such as monoclonal antibodies^16^. Recent studies have begun exploring MAM workflows for more complex biologics, including fusion proteins and glycoproteins with multiple glycosylation sites^12,15^. MAM on proteins with a higher number of glycosylation sites or with high glycan heterogeneity such as subunit vaccine molecules is challenging. The higher the number of glycosylation sites on a protein, the greater the sample quantity requirements, processing requirements, complexity and time of analysis^17^. We developed MAM to support process development of a multimeric influenza nanoparticle vaccine and an HIV trimer envelope vaccine. We performed detailed structural characterization of the subunit protein vaccines and monitored critical parameters such as glycosylation, protein chain clipping, deamidation and oxidation of methionine—designated as stage 1 of MAM. Consequently, we developed a prototype routine high throughput workflow for monitoring the potential critical attributes of vaccine therapeutics to support process development and manufacturing by MAM—stage 2 of MAM. Our study demonstrates the applicability of MAM to highly complex glycosylated multi-subunit vaccine molecules throughout early stages of process development, from cell line development to formulation development and stability. By integrating structural characterization with process monitoring, we aim to bridge the gap between early analytical development and scalable QC implementation.

## Materials and methods

### MAM workflow development

A prototype MAM workflow was developed to evaluate multiple attributes of heavily glycosylated protein subunit vaccine molecules with higher levels of heterogeneity due to glycosylation. The detailed characterization of vaccine subunits for chain clipping, glycosylation sites, glycosylation profile, oxidation and deamidation were conducted using LC–MS/MS (peptide mapping and subunit analysis). Data were generated on the following MS systems: Orbitrap Q Exactive MS with Waters Acquity H-Class UPLC-system; Orbitrap Exploris 480 MS with Ultimate 3000 LC-system; and Waters Xevo G2-XS MS with Waters Acquity H-Class UPLC-system. The data processing was performed by using Thermo Fisher Scientific Xcalibur (version 4.1; https://www.thermofisher.com/us/en/home/industrial/mass-spectrometry/liquid-chromatography-mass-spectrometry-lc-ms/lc–ms-software/lc-ms-data-acquisition-software/xcalibur-data-acquisition-interpretation-software.html) Thermo Fisher Scientific BioPharma Finder (version 5.0; https://www.thermofisher.com/us/en/home/industrial/mass-spectrometry/liquid-chromatography-mass-spectrometry-lc-ms/lc-ms-software/multi-omics-data-analysis/biopharma-finder-software.html), Protein Metrics Byos software (version 5.8; https://www.proteinmetrics.com/products/byos)—peptide mapping module and intact mass modules, and Waters MassLynx (Version 4.2; https://support.waters.com/KB_Inf/MassLynx).

### In solution protease digestion

About 100 µg protein was dissolved in 25 µL of 50 mM aq. NH_4_HCO_3_. To this 1.5 µL of 500 mM dithiothreitol (DTT) (dissolve the DTT with 100 µL of 50 mM aq. NH_4_HCO_3_) was added and kept at 45 °C for 20 min. 4.5 µL of 500 mM Iodoacetamide (IAA) (dissolve the IAA with 100 µL of 50 mM aq. NH_4_HCO_3_) was added to the samples and kept at room temperature (RT) in dark for 15 min. After incubation, the samples were desalted with 10 kDa centrifuge filters (Millipore Sigma Amicon Ultra-0.5). The sample was recovered, and the volume of sample was adjusted to 50 µL using 50 mM aq. NH_4_HCO_3_. The protein sample was digested by adding 2 µL sequencing-grade trypsin (Promega, 2.5 µg/µL) (1:20 ratio) and incubated at 37 °C for 12 h. The protease was inactivated by adding 1 µL of 1% formic acid. The peptides were diluted with 0.1% formic acid in water to 1 µg/µL concentration and stored at − 30 °C until analysis by LC–MS/MS.

### Cell culture and clone generation

Our CHO-DG44 cell line was obtained from Dr. Lawrence Chasin at Columbia University and was adapted to serum-free suspension culture at the Vaccine Production Program Laboratory (VPPL), Vaccine Research Center (VRC), National Institute of Allergy and Infectious Diseases (NIAID), National Institutes of Health (NIH). Stable cell pools were generated by MaxCyte electroporation of CHO-DG44 cells with an expression vector containing the dihydrofolate reductase selection marker and the coding sequence for the Hemagglutinin (HA) stabilized stem construct. Following selection with methotrexate, the recovered stable pools were subjected to single cell cloning using the VIPS (Advanced Instruments). The clonally derived cells lines were scaled up from 96-well plates to shake flasks based on growth and productivity. Top clones were cultured in a 14-day fed-batch in ActiPro medium supplemented with Cell Boost 7a and 7b (Cytiva) to promote cell growth and protein expression. Cell culture harvest was clarified by centrifugation at 3000 rpm for 30 min, decanted, and filtered using a 1L sterile disposable filter unit with a 0.2 µm PES membrane.

### Downstream purification of proteins

CHO DG44 cells expressing the protein of interest were clarified by a multi-step filtration train. The filtered material was subjected to detergents for the inactivation of adventitious and endogenous viruses, then purified further by bind and elute chromatography with anion exchange resin (AEX). The eluted material was concentrated, and buffer exchanged utilizing tangential flow filtration (TFF 1) which was further purified using flow-through chromatography (Mixed-mode chromatography (MMC)) employing a polishing resin for the removal of any remaining impurities. The flow-through sample was nano-filtered to remove any endogenous viruses and finally buffer exchanged and concentrated with another tangential flow filtration step (TFF 2). The product of each unit operation was sterile filtered to minimize possible contamination during execution of each unit operation.

### Stability assessment of lead formulation buffers

HIV envelope trimer vaccine was formulated in the candidate formulation buffers at 2 mg/mL. Formulated samples were sterile filtered and aseptically vialed in clinically analogous container-closure systems, and stored at ≤ − 60 °C, − 20 °C ± 5 °C, 5 °C ± 3 °C, 25 °C ± 2 °C and 40 °C ± 2 °C for up to 8 weeks. Samples were tested at pre-defined timepoints by a panel of characterization assays. Buffer compositions were as follows: For S1 and S2—formulation (A)—10 mM Sodium Phosphate, 10 mM NaCl, 7.5% (w/v) Sorbitol, 0.01% (w/v) Polysorbate 80, pH 7.2; for S3—formulation (B) 10 mM Sodium Phosphate, 10 mM NaCl, 7.5% (w/v) Sorbitol, 0.01% (w/v) Polysorbate 80, 5mM L-Methionine, pH 7.2.

### Cell culture supernatant in-gel protease digestion

The cell culture supernatants with expressed HA stem vaccine components were fractionated on separate lanes using SDS-PAGE. The gel was stained by Coomassie dye and the bands corresponding to HA vaccine component were cut (based on western blotting results) into smaller pieces (1 mm squares approx.) and transferred to clean tubes. The gel pieces were destained by adding 100 μL acetonitrile (ACN): 50 mM NH_4_HCO_3_ (1:1) and incubated at RT for about 30 min. Tubes were centrifuged, the supernatant was discarded and 100 μL ACN was added before incubation for 30 min. The proteins on gel pieces were reduced by adding 1.5 μL 500 mM DTT in 300 μL NH_4_HCO_3_ and incubating at 45 °C for 20 min. The tubes were cooled to RT and the supernatant was removed. The gels were washed with 300 μL of ACN, added 4.5 μL 500 mM iodoacetamide in 300 μL NH_4_HCO_3_ and the mixture was incubated at RT for 15 min in the dark. Proteins were digested by adding 1 μLsequence-grade trypsin (Promega, 0.5 µg/µL) in digestion buffer (50 mM NH_4_HCO_3_) for 12 h at 37 °C. The peptides were extracted out from the gel by addition of 1:2 H_2_O:ACN containing 5% formic acid (300 μL), and the released peptides were dried under speed vacuum. The samples were reconstituted in aqueous 0.1% formic acid for LC–MS/MS experiments^18^.

### Deglycosylation of protein subunit for intact mass analysis

The proteins samples were subjected to reduction and subsequent deglycosylation by the treatment of both Rapid PNGaseF (New England Biolabs (NEB)) and regular PNGaseF (Promega) sequentially at 50 °C and 37 °C, respectively, in Rapid PNGaseF buffer for complete deglycosylation before intact subunit mass analysis. Briefly, about 16 µg of each sample was taken in a microcentrifuge tube. The volume was adjusted to 8 µL with water. 2 µL of Rapid PNGaseF buffer was added to each sample and heated to 80 °C for 5 min for denaturation. Further, 2 µL of Rapid PNGaseF was added to each sample and incubated at 50 °C for 2 h. After the incubation, 2 µL of regular PNGaseF was added to each sample and incubated at 37 °C for overnight.

### Data acquisition for intact subunit analysis

The volume of the samples after deglycosylation was adjusted to 50 µL with 0.1% formic acid and injected 5 µL into a Waters Xevo-G2-XS LC–MS system with a C4 column (Waters S/N: 186004496) at 80 °C. LC mobile phase A was water with 0.1% formic acid and mobile phase B was acetonitrile with 0.1% formic acid and the gradient at a flow rate of 0.3 mL/min was: 10% B at 0 min, 10% B at 3 min, 25% B at 3.1 min, 45% B at 33 min, 95% B at 33.1 min, 95% B at 36 min, 10% B at 36.1 min, 10% B at 40 min. The LC–MS data were processed through MassLynx software (MaxEnt 1) and Byos intact mass analysis module.

### Data acquisition of protein digest samples using LC–MS/MS

The peptides were analyzed on an Orbitrap Exploris 480 or Q Exactive HF LC–MS/MS system by injecting about 1 to 3 µg (1 to 3 µL) into the C18 column (Waters S/N: 186002350) at 45 °C per injection. LC mobile phase A was water with 0.1% formic acid and mobile phase B was acetonitrile with 0.1% formic acid and the gradient at a flow rate of 0.2 mL/min was: 3% B at 0 min, 3% B at 1 min, 50% B at 28 min, 95% B at 29 min, 95% B at 32 min, 3% B at 33 min, 3% B at 38 min. For runs on the Q Exactive HF, after the precursor ion scan at 120,000 resolution in Orbitrap analyzer, top 5 precursors were selected for subsequent fragmentation using higher-energy collisional dissociation at normalized collision energy of 28. The fragment ions were analyzed on Orbitrap analyzer at 30,000 resolution. Dynamic exclusion was enabled with an exclusion duration of 10 s. For runs on the Orbitrap Exploris 480, after the precursor ion scan at 120,000 resolution in Orbitrap analyzer, precursors were selected for 3 s for subsequent fragmentation using HCD at normalized collision energy of 30. The fragment ions were analyzed on Orbitrap analyzer for HCD at 45,000 resolution. Dynamic exclusion was enabled with an exclusion duration of 20 s.

### Peptides mapping data analysis

The .raw files from the LC–MS/MS acquisition peptides were analyzed through Byos peptide mapping software by searching against the fasta sequence of the corresponding protein. The search was conducted with modifications such as carbamidomethylation of cysteine (fixed modification), oxidation, dethiomethyl, and formylation of methionine, and deamidation of asparagine. A precursor ion tolerance of 5 ppm and fragment ion tolerance of 10 ppm was set for the search with up to two missed cleavages for the target enzyme trypsin. 182 most common human N-glycans were used as a variable modification for N-glycosylation searches. Based on the identifications of the software (after manual validation of spectra), the sequence of the peptide and PTM was determined and quantified using Byos software.

## Results

### New generation vaccines and monitoring critical vaccine attributes by MAM

**Fig. 1:**
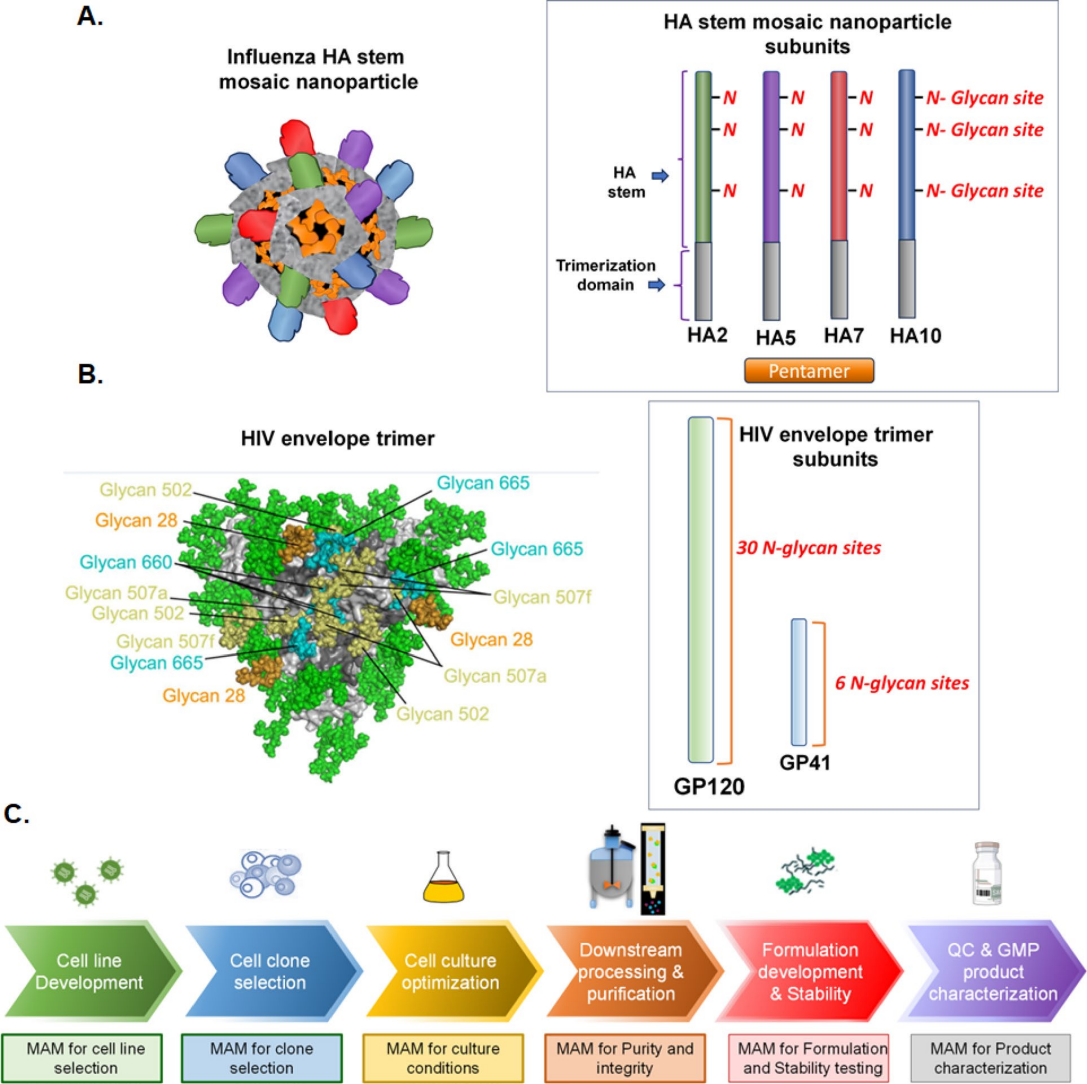
Workflow for MAM integration into heavily glycosylated subunit vaccine process development. MAM development focused on (**A**) Influenza Hemagglutinin stem mosaic nanoparticle vaccine subunits (three N-glycosylation sites in each subunit) and (**B**) HIV envelope trimer^19^ (30 and 6 N-glycosylation sites, respectively in GP120 and GP41). (**C**) Stages of vaccine process development for which MAM was developed. The figure illustrates how MAM enables high throughput monitoring of glycosylation and other critical quality attributes across multiple stages of vaccine production.

Two vaccines with extensive glycosylation were chosen for MAM development and proof of concept process integration studies. The influenza nanoparticle vaccine (also known as SteMos1) contains a predefined ratio of HA stem domains from the different strains (Fig. 1A). Each HA stem component has three N-glycosylation sites adorned with complex, hybrid, and oligomannose type N-glycans (Fig. 1A), for a total of twelve unique glycosylation sites with highly heterogeneous glycan profiles. The HIV trimer vaccine (also known as Trimer 7678) includes 30 potential N-glycosylation sites on the GP120 subunit and six potential N-glycosylation sites on the GP41 subunit of each heterodimer unit (Fig. 1B)^19^.

MAM was evaluated for characterizing PTMs at each stage of development (Fig. 1C) for components of the two vaccines (Table 1).

**Table 1:** Potential critical attributes of vaccine subunits monitored by MAM analysis. Evaluating these attributes are essential for ensuring product consistency, safety, and efficacy during vaccine development.

Monitored attributes in MAM experiments
1. Intact protein subunit mass analysis
2. Protein sequence coverage
3. N- and C-terminal clipping and protein cleavage
4. Quantitative profiling and comparison of Glycosylation on multiple N-glycosylation sites
5. Glycosylation site occupancy
6. Deamidation on asparagine
7. Methionine oxidation
8. HCP identification and quantification

### PTM comparison of influenza vaccine components expressed by different cell lines and application of MAM to cell clone selection

**Fig. 2:**
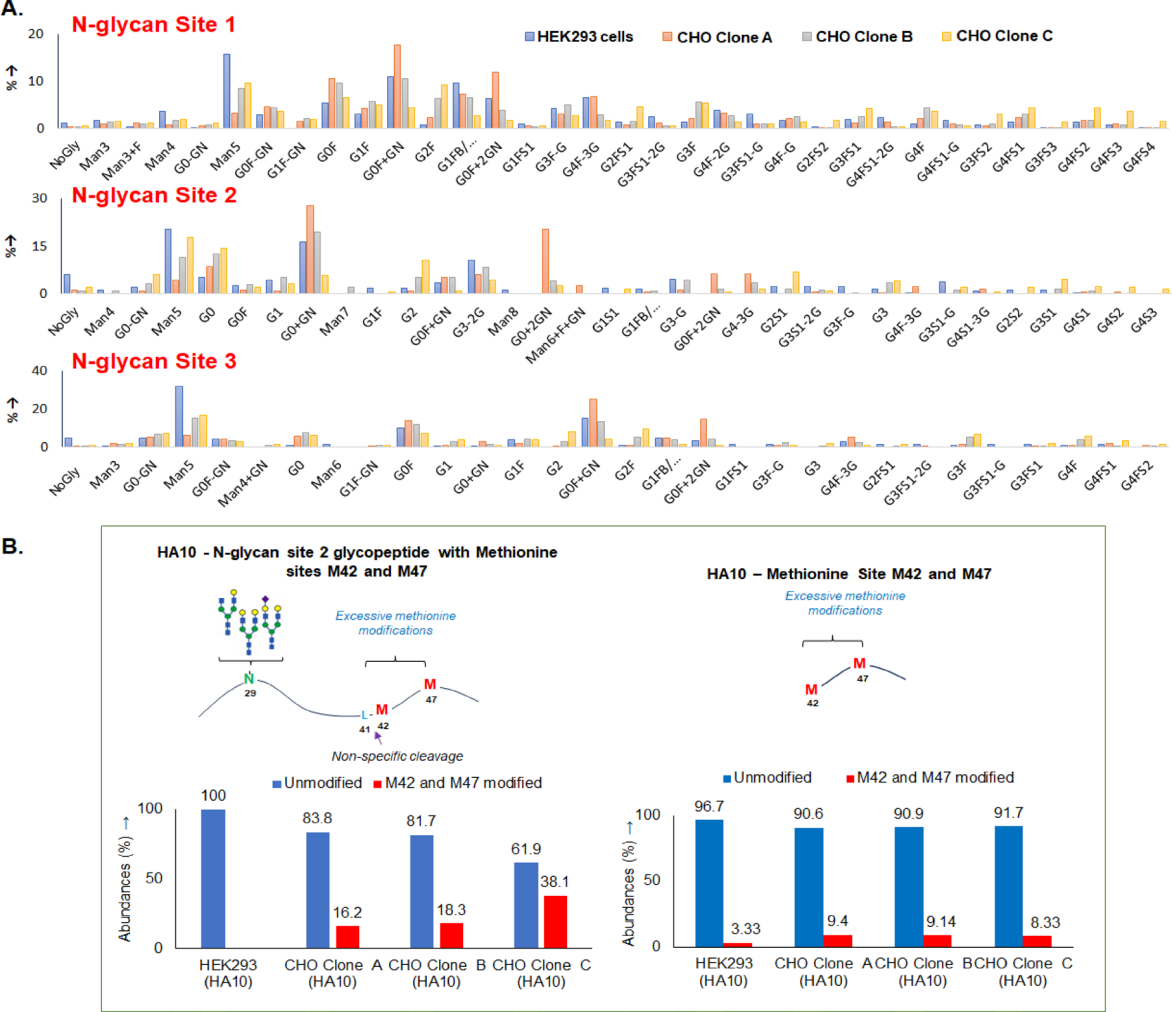
HA10 stem component expressed from HEK293 cells and three CHO clones were compared using MAM. (**A**) N-glycosylation on HA10 expressed in HEK293 cells and three clones of CHO. (**B**) Modifications (oxidation and dethiomethylation) on methionines 42 and 47 of HA10 expressed in HEK293 cells and three clones of CHO, non-specific cleavage of peptide and generation on shorter peptide with different methionine modification was also observed. These results highlight the capability of MAM to resolve subtle differences in post-translational modifications and glycosylation patterns across different expression systems, which are critical for vaccine consistency and quality.

Individual HA stem proteins were initially expressed in HEK293 cells during vaccine discovery and then expressed by CHO cells for process development and manufacturing. Proteins expressed in HEK293 and CHO cells were compared for sequence identity, quantitative glycosylation profiling at three N-glycosylation sites, and other common PTMs such as methionine oxidation and asparagine deamidation. Purified H10 HA stabilized stem components (HA10) expressed in HEK293 cells were compared with partially purified HA10 (after anion exchange chromatography (AEX)) expressed in three different CHO cell lines, showing quantitative differences in PTMs. HA10 CHO clone B contained similar levels of the most abundant glycans (Man5 and G0F + GN) at both sites 1 and 3, and similar levels of Man5 and G0 + GN for site 2, in comparison to HEK293 expressed protein (Fig. 2A). Interestingly, oxidation of proximal methionines 42 and 47 was observed for HA10 expressed in CHO, which was not observed in the HEK expressed version (Fig. 2B). The combined oxidation content ranged between 16 and 38%, with clones A and B having much lower content than clone C. Based on the evaluation of these attributes, the CHO clone closely matching the HEK-expressed components (clone B) was chosen for further product development. Similar data were generated for the other three HA stem components, HA2, HA5, and HA7 (Supp. Table 1, Supp. Figures S1 to S23). These baseline data on cell line differences in PTMs demonstrated the feasibility of MAM for highly glycosylated proteins and its potential utility for selecting cell lines and clones for product development.

**Fig. 3:**
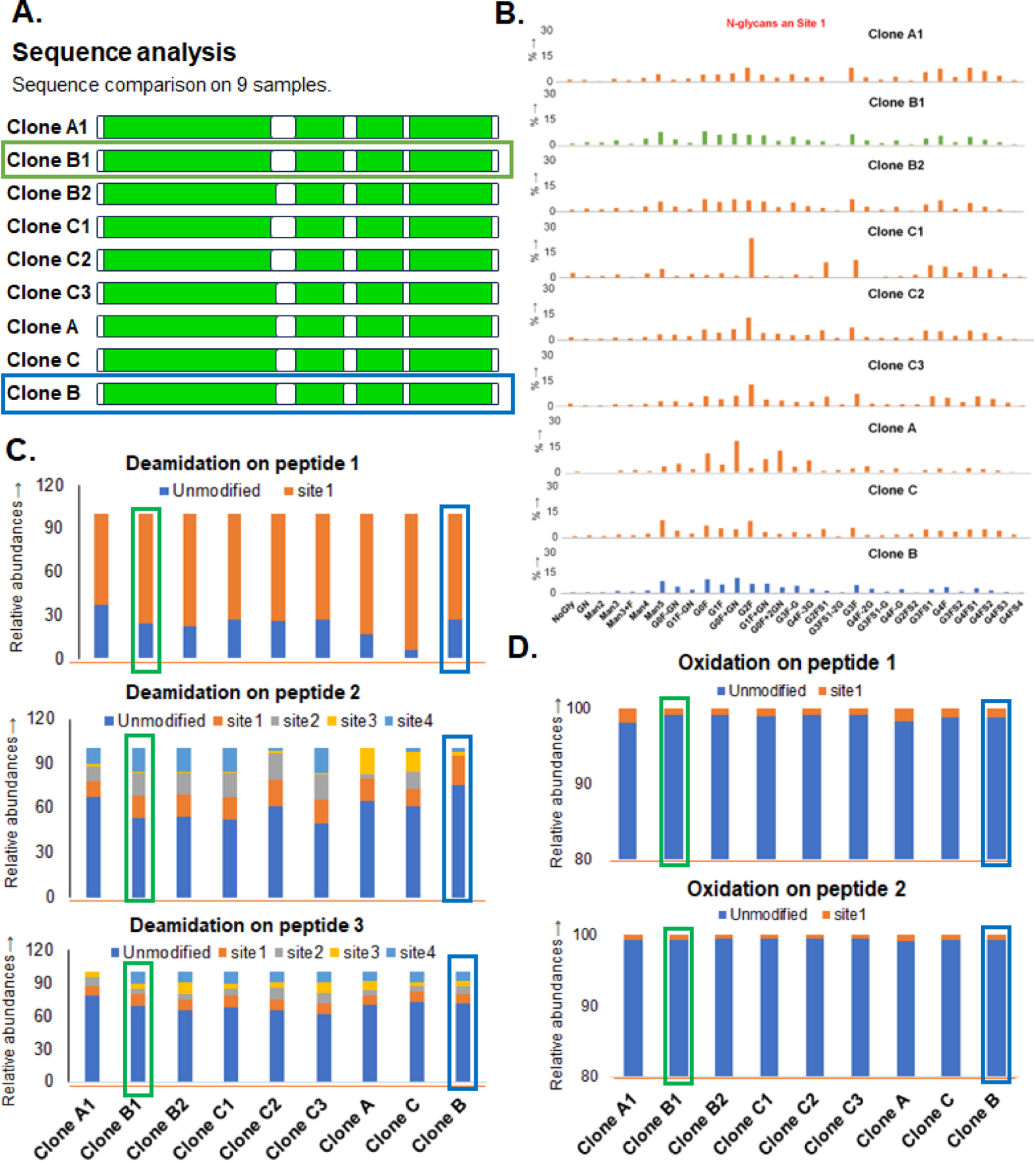
MAM analysis of CHO-expressed hemagglutinin stem HA10 under different bioreactor conditions. (clones A, B and C; bioreactor conditions 1, 2 and 3) (**A**) Sequence comparison (amino acid sequence coverage is highlighted in green); (**B**) Comparison of N-glycosylation at site 1 of HA10 stem protein; (**C**) Asparagine deamidation on three peptides; (**D**) Methionine oxidation on two peptides. This figure demonstrates how MAM can be used to assess the impact of bioreactor conditions on critical quality attributes, enabling fine-tuning of process parameters for improved product quality.

Bioreactor growth studies were conducted with the three CHO cell clones and expressed HA stem protein was assessed by MAM for protein sequence identity, protein truncation, glycosylation at individual sites, deamidation and oxidation profiles, and host cell protein contamination (HCP)^5^. Identical sequence coverage of HA10 was obtained for proteins from the HA10 clones (clones A, B and C) in all growth conditions (conditions 1, 2, and 3) but varying profiles of N-glycans were observed on three sites of the protein (Fig. 3A, B, Supp. Figure S26-S27). Similar levels of asparagine deamidation and oxidation were observed for the different clones and conditions (Fig. 3C, D). Condition B1 HA10 protein had similar glycosylation and other PTM profiles with the HA10 from the control condition for clone B, which had similarity with HEK293 expressed HA10. Precision in the quantification of PTM such as glycosylation, deamidation and oxidation during the sample preparation, LC–MS data acquisition and software processing were evaluated by performing triplicate of HA10 digestions and triplicate of LC acquisitions (Supp. Figures S28-S29). Thus, clone B with conditions B1 was chosen for further development. Similar screening was conducted for other HA strains (HA2, HA5, and HA7) and optimum clones and culture conditions were selected for further development (Supp. Figures S1-S23, Supp. Table S1, Supplementary material 2 (Excel file) contains the full peptide mapping data).

We also evaluated the HCP profiles of purified HA10 and identified the most common coeluting contaminant proteins. Proteins such as protein disulfide isomerase and 14-3-3 protein isoforms were among the most common HCP detected in all clones (Table 2). ELISA-based CHO HCP screening kits have limitations in detecting certain HCPs because they only target immunogenic proteins. Some HCPs might not be detected due to weak antibody binding or poor assay conditions^20^. MAM analysis may therefore be a critically useful tool to broadly identify HCPs in purified protein preparations as well as process steps, and to guide the design of strategies to mitigate HCP impurities.

**Table 2:** Comparison of host cell proteins detected on HA10 isolated from three CHO cell clones under different bioreactor conditions (Values are percentage of total protein detected; ND- not detected).

Protein	Clone A		Clone B			Clone C			
	Baseline	1	Baseline	1	2	Baseline	1	2	3
HA10 stem	98.6	97.9	99.7	99.0	99.0	99.4	99.2	99.2	99.2
Vimentin	0.1	0.7	ND	0.4	0.4	0.1	0.4	0.4	0.4
Dickkopf-related protein 3 isoform X1	0.1	0.3	0	0.1	0.1	0	ND	0.2	0.1
14–3-3 protein epsilon	0.3	0.2	0.2	0.2	0.1	0.2	0.1	0.1	0.1
Protein disulfide-isomerase	0.1	0.2	ND	ND	ND	0.1	ND	ND	ND
Protein disulfide-isomerase A6	0.1	0.1	ND	0.1	0.1	0.1	ND	0.1	ND
Nucleobindin 2	ND	0.1	ND	ND	ND	ND	ND	ND	ND
Tropomy in 4	0.2	0.1	ND	ND	ND	ND	ND	ND	ND
Nidogen-1	0	0.1	ND	ND	ND	ND	ND	ND	ND
SPARC	0.2	ND	ND	ND	ND	ND	ND	ND	ND
14-3-3 protein zeta/delta	ND	ND	ND	ND	0.1	ND	ND	ND	ND
Protein disulfide-isomerase A4	0.1	ND	ND	ND	ND	ND	ND	ND	ND
Dickkopf WNT signaling pathway inhibitor 3	ND	ND	0.1	ND	ND	0.1	0.1	ND	ND

This analysis highlights clone- and process-dependent variability in host cell protein profiles, which can influence product purity and downstream processing requirements.

### Cell culture and harvest optimization by MAM evaluation of culture supernatants

**Fig. 4:**
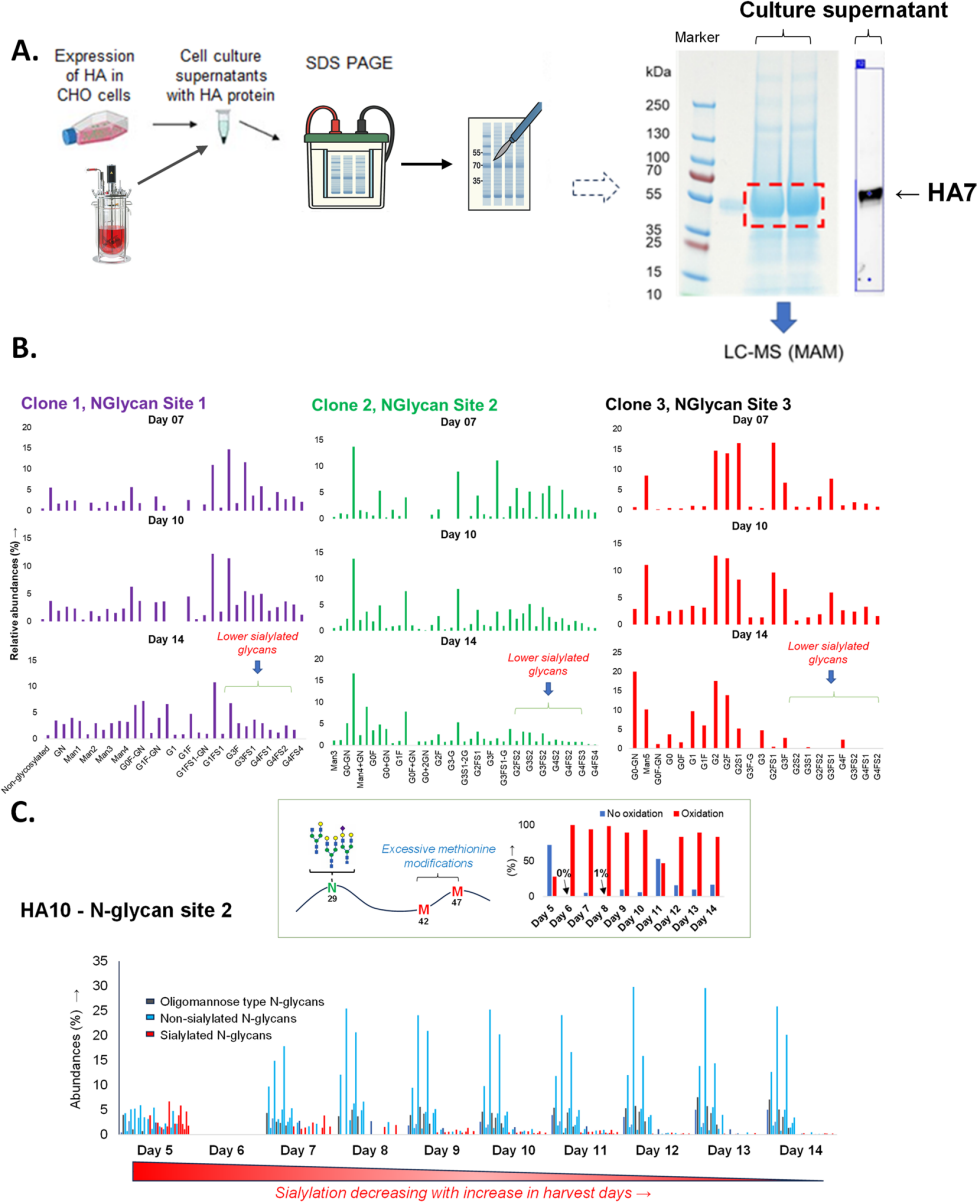
CHO cell harvest optimization through MAM. (**A**) Process flow for direct analysis of cell culture supernatant via separation on SDS gel and processed by in-gel digestion guided through western blotting; (**B**) HA7 stem (Clone A) protein N-glycosylation and sialylation at multiple days of culture; (**C**) Sialylation and methionine oxidation (inset) of HA10 throughout 14 days of culture. This demonstrates how MAM enables monitoring of post-translational modifications directly from harvested supernatants, providing insights into culture-dependent changes in glycosylation and oxidation that can inform upstream process optimization.

Evaluation of the effect of cell culture conditions (time, temperature, pH etc.) on expressed protein and associated PTMs is limited by the need to generate a purified sample of a low abundant protein from the culture sample. We addressed this challenge by a simple but robust method of directly analyzing cell culture supernatants. This method enables characterization of the protein vaccine and its PTMs from different clones, culture conditions and protein harvest time points by MAM. Cell culture supernatants were loaded directly onto SDS gels without purification, and the proteins from the expected molecular weight region, determined based on western blotting, were excised for in-gel protein digestion and LC–MS/MS (Fig. 4A). The data from multiple clones and harvest conditions were analyzed and quantified by MAM; allowing for comparison of PTMs, such as glycosylation profiles at N-glycosylation sites, deamidation, and oxidation profiles.

Analysis of the HA7 stem protein expressed in three CHO cell clones and sampled on day 7, 10, or 14 of fed-batch culture showed a similar time-dependent trend for the glycosylation profile across all three N-glycosylation sites. Notably, sialylation decreased as the culture days progressed. Marginal elevation of high mannose glycans was also observed at day 14, relative to the earlier time points (Fig. 4B).

We also evaluated the glycosylation profile and PTM changes of HA10 stem protein over time. As with HA7, increased days of culture reduced the overall sialyation on the N-glycans on all three N-glycosylation sites of the protein (Fig. 4C). Surprisingly, no glycopeptides with only N-glycosylation modification at site 2 of HA10 were detected on day 6. Further investigation showed complete modifications of two methionines in the glycopeptide at day 6, shifting the mass of glycopeptides. Moreover, increased days of culture induced excessive oxidation of methionines, leading to generation of oxidized, dethiomethylated, and formylated methionine species (Fig. 4C inset, Supp. figures S24-S25, S30-S38). However, day 11 showed reduced oxidation in comparison to other time points and this was confirmed in multiple sample acquisitions. This indicates the possibility of oxidation during the process of culture harvest and sample processing. Taking these data together, the MAM-based method of analyzing unpurified cell culture samples provided robust characterization of PTM of the highly glycosylated protein of interest.

### MAM guided downstream processing and purification optimization of vaccines

**Fig. 5:**
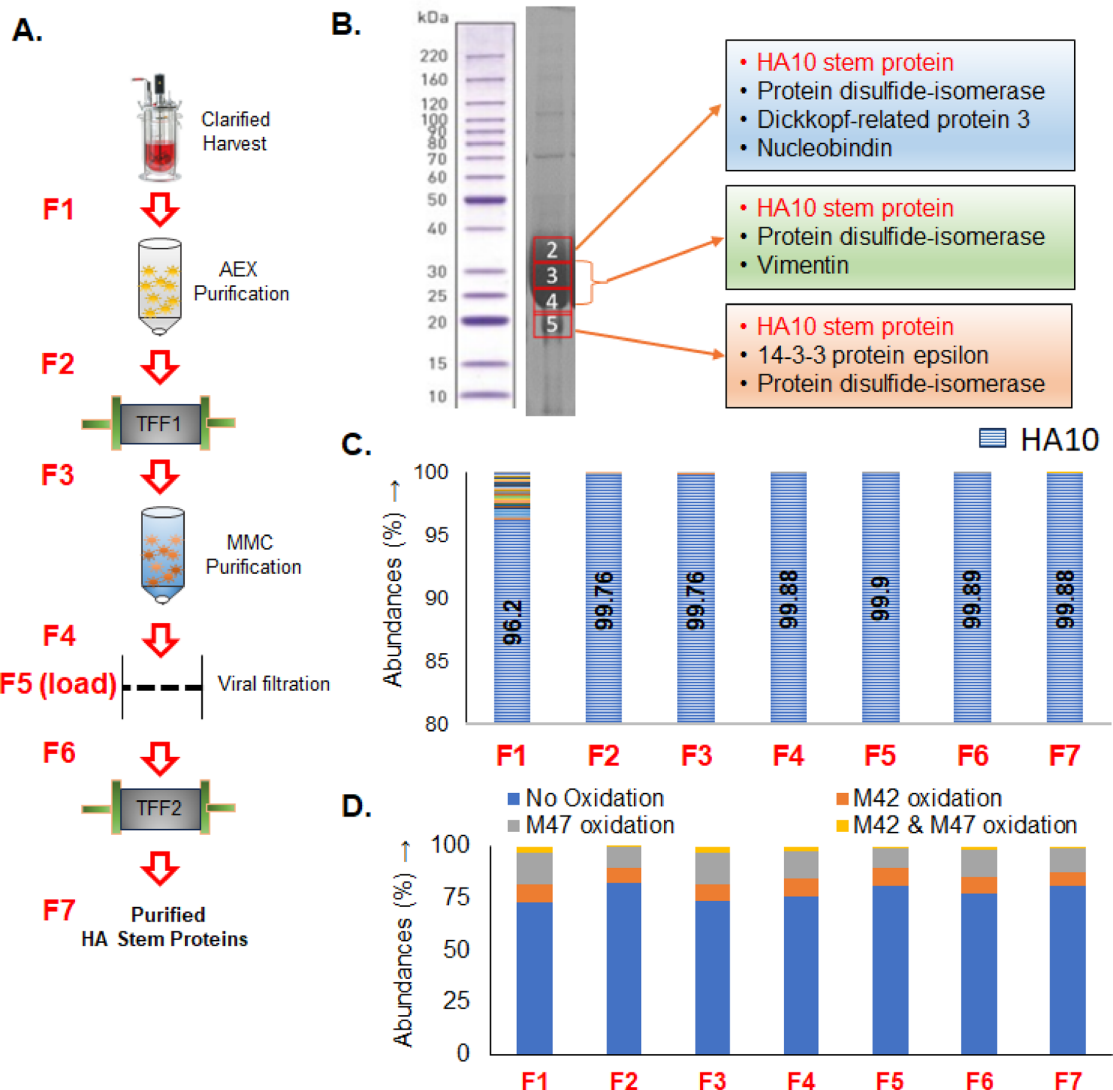
Monitoring downstream purification for PTM and HCP changes using MAM, (**A**) Downstream process for the purification of HA stem proteins. (**B**) HCPs identified on purified HA10 stem protein at upper, middle and lower sections of protein band by in-gel protein digestion followed by MAM (highest abundant proteins were shown) (**C**) HCP analysis during stages of HA10 protein purification. (**D**) Monitoring oxidation of methionine on HA10 stem protein at different stages of proteins purification; AEX—anion exchange chromatography, MMC—mixed mode chromatography. This highlights the utility of MAM in tracking product quality attributes and impurity profiles throughout downstream processing, enabling data-driven optimization of purification strategies.

The PTM and HCP profile of a vaccine can be altered during purification processes as subpopulations are removed; purified material also has the potential for chemical degradation as it is subjected to changing buffers and environments. To determine if MAM analysis can identify changes (or lack thereof) during purification, critical attributes of each HA stem component were monitored by MAM during each step of the downstream purification process (Fig. 5A, Supplementary material 3 (Excel file) contains the HCP data).

The most notable changes observed for the HA10 stem protein were methionine oxidation and HCP abundance. Oxidation of methionine observed at the cell harvest step remained consistent throughout the process, indicating that purification process neither induced further oxidation nor removed the oxidated species (Fig. 5D). Additional investigations are required to study the effect of methionine modifications on the physicochemical and functional properties of the HA10. During the screening of HCPs on the purified HA10 stem component at different stages of product purification by MAM, HCPs coeluting with the products were identified (Fig. 5C, Supplementary material 3 (Excel file) contains the HCP data). To monitor the abundance of the primary HCPs coeluting after anion exchange purification (F2 in Fig. 5), an in-gel digestion was conducted of the HA10 band divided into upper, middle and lower sections (Fig. 5B). Interestingly, different types of HCP were detected in each section with protein disulfide isomerase and its isomers as most common HCP present in all three sections (Fig. 5B) and nearly all HCP were removed by the anion-exchange chromatography step (Fig. 5C). This data could guide future refinement of process parameters to mitigate undesired PTMs and target removal of specific HCPs that may affect vaccine quality or efficacy.

### Intact-MAM and MAM monitoring of protein degradation

During development of a clinical formulation for the HIV trimer subunit vaccine, a stability study was conducted to evaluate stabilizing effect of lead buffers under stress, including the effect of L-methionine addition to minimize oxidative degradation. To determine if MAM could be used to enhance understanding of vaccine degradation and buffer stabilization during the formulation development process, detailed LC–MS studies were conducted, including both intact subunit mass analysis and peptide mapping analysis. The analysis primarily focused on the N-glycosylation of selected sites of the vaccine subunits previously shown or suspected to be critical to function, as well as oxidation and deamidation of selected methionine and asparagine residues^19^.

Although the MAM workflow typically includes monitoring critical product attributes at the peptide level, some critical information cannot be monitored at this level, such as assembly of individual protein subunits, the presence of protein chain clipping, or aggregate formation. Recent studies have reported the use of a combination intact protein mass spectrometry and the multi-attribute method which is termed as intact multi-attribute method—or iMAM^11^. This modified MAM method was applied to the HIV trimer subunit vaccine to evaluate the extent of protein chain clipping in select stability samples. Detailed intact subunit mass analysis was conducted by optimizing protein deconvolution parameters. A set of heat stressed samples in phosphate buffer saline (PBS) were generated to understand chain cleavages and the intact masses of samples were compared by monitoring masses of deglycosylated subunits (Supp. Figure S39). Chain truncation towards the N-terminal of GP120 subunit and C-terminal of GP41 subunit was observed.

**Fig. 6:**
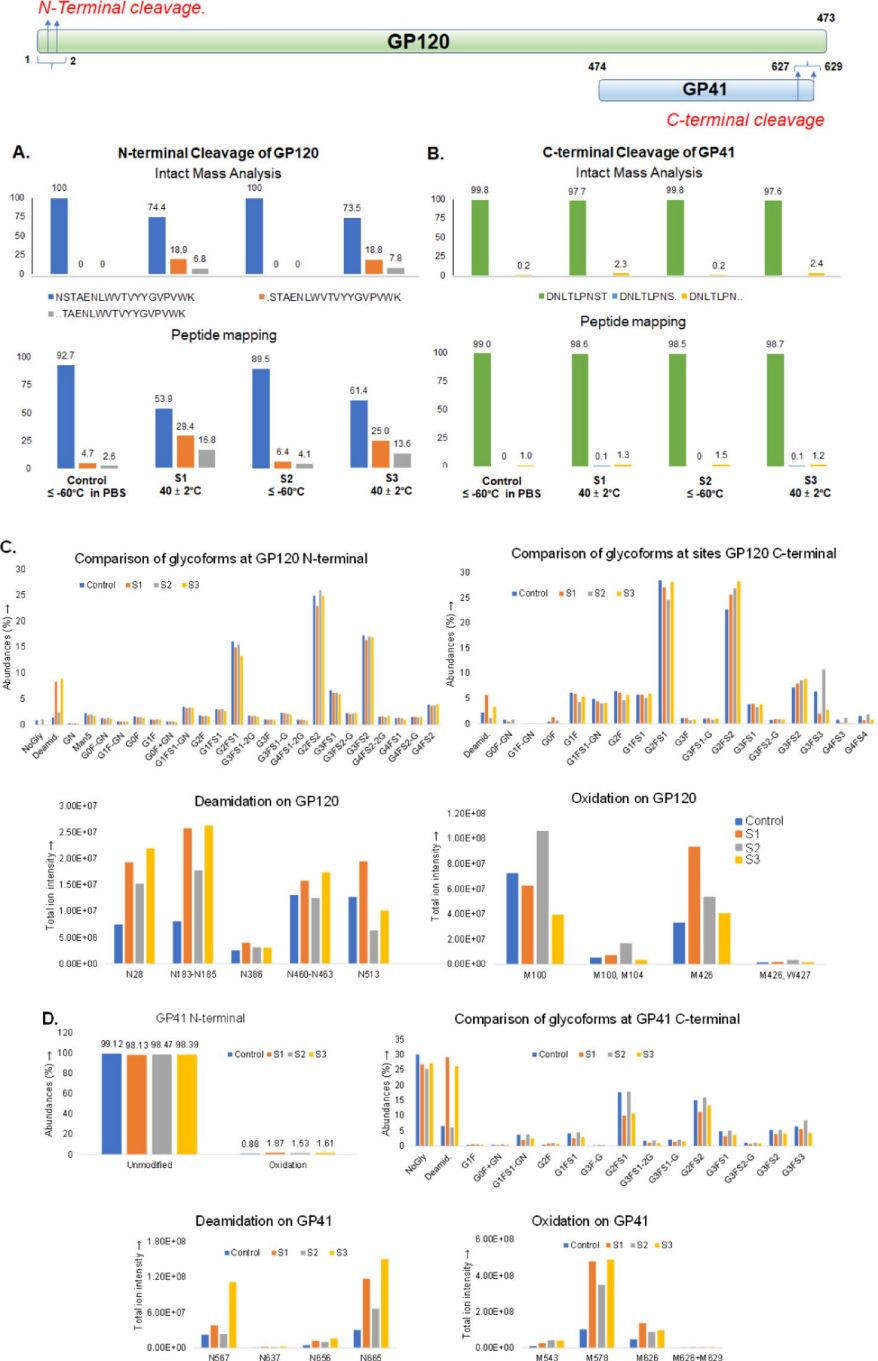
Analysis of stability study batches of HIV trimer GP120 and GP41 subunits for N- and C-terminal cleavage determination and PTM comparison by intact mass and peptide mapping analysis. Control ≤ − 60 °C in PBS; formulation A stored at 40 ± 2 °C for 2 weeks (S1); formulation A stored at ≤ − 60 °C for 4 weeks (S2); and formulation B stored at 40 ± 2 °C for 4 weeks (S3). Evaluation of chain truncation by intact and peptide mapping on A. GP120 and B. GP41 subunit. MAM peptide mapping analysis for PTM comparisons C. GP120 subunit, D. GP41 subunit. The impact of formulation and storage conditions on protein integrity and PTM profiles can be monitored by MAM and it provides insights into the stability of HIV vaccine candidates under accelerated and long-term storage conditions.

Subsequently, another set of samples were analyzed by intact mass analysis: control (stored at ≤ − 60 °C in PBS); formulation A, stored at 40 ± 2 °C for 4 weeks (S1); formulation A stored at ≤ − 60 °C for 4 weeks (S2); and formulation B stored at 40 ± 2 °C for 4 weeks (S3) (Fig. 6). Analysis of the intact subunits indicated multiple changes occurred in the temperature stressed samples in different formulations. The intact subunit mass analysis of GP120 subunit was similar for the control and sample S2 (53,520 Da), while the mass of stressed samples S1 and S3 were 53,524 Da and 53,522 Da, respectively, suggesting elevated protein deamidation (Supp. Figure S40A). Spectra of S1 and S3 also contained new peaks at around 53,405 Da and 53,320 Da, indicating sequential loss of N-terminal residues of GP120. Intact mass of the cleaved products suggests that cleavages are happening on the first two residues of the N-terminus of GP120, amino acids asparagine N^1^ (glycosylation site) and serine S^2^ (Fig. 6A). No other cleaved protein masses were observed in stressed samples which suggests that no further peeling of residues or loss of glycosylation was observed on the GP120 subunit. Peptide mapping analysis, with quantification of N-terminal peptides ‘N^1^S^2^T^3^AENLWVTVYYGVPVWK’ of GP120 and its cleaved fragments ‘S^2^TAENLWVTVYYGVPVWK’ and ‘T^3^AENLWVTVYYGVPVWK’, shows elevated levels of the cleaved fragments in stressed samples, supporting the conclusions of the iMAM analysis (Fig. 6A).

Intact subunit mass analysis of the GP41 subunit confirmed a similar mass of 17,510 Da for all samples (Supp. Figure S40B). However, increased intensity of fragment peaks was observed in samples S1 and S3 (17,322 Da), indicating a loss of C-terminal residues of GP41 (Supp. Figure S40B). The intact mass of cleaved products suggests that cleavages are happening on the last two C-terminal residues of GP41 in samples S1 and S3, amino acids serine S^634^ and threonine T^635^ (Fig. 6B), as a peak corresponding to the combined loss of serine and threonine can only be generated by a cleavage at C-terminal of GP41. No other cleaved protein masses were observed in stressed samples which suggests no further peeling of residues or loss of glycosylation on the GP41 subunit. The N-terminal peptide ‘A^1^VGLGAVFLGFLGAAGSTMGAASNTLTVQAR’ of GP41 was monitored for the cleavage products and no cleavage of residues was observed on the N-terminal peptide of GP41 in stressed samples in comparison to the control and S2 (Fig. 6D). Monitoring the C-terminal peptide ‘DNLTLPNST’ of GP41 with two sites of glycosylation showed generation of cleaved fragments ‘DNLTLPNS’ and ‘DNLTLPN’ in the stressed samples as observed in iMAM analysis (Fig. 6B). Increased loss of C-terminal end residues of GP41 was observed on stressed samples S1 and S3 in comparison to control and S2 (Fig. 6B).

The HIV trimer GP120 subunit contains 30 potential glycosylation sites; select sites were chosen for MAM PTM analysis based on postulated functional and/or stability impact^19^. The N-terminal peptide ‘N^1^STAENLWVTVYYGVPVWK’ of GP120 subunit contains N-glycosylation on first residue (labelled as N^1^). No significant PTM changes were observed at site N^1^, with the exception of increased deamidation in samples S1 and S3 (Fig. 6C). Analysis of GP120 C-terminal peptide ‘TVVENSTHKNLTHHM’ (two sites of N-glycosylation) showed no peeling of residues or loss of glycosylation in any of the tested samples (Fig. 6C). Similar to the N-terminal peptide, however, increased deamidation was observed on the C-terminal peptide in samples S1 and S3 in comparison to control and S2, although no significant changes were observed in the overall glycosylation at the two N-glycosylation sites except for the minor loss of tri and tetra antennary sialylated glycans (Fig. 6C). Comparison of deamidation at different sites of GP120 (peptides without N-glycan consensus sequence) revealed overall increased deamidation in stressed samples in comparison to control and S2 (Fig. 6C). The oxidation levels on selected methionine sites of GP120 were elevated at different sites but, expectedly, the oxidation of methionine was less in S3 in comparison to other samples suggesting that addition of methionine in the S3 formulation reduces methionine oxidation (Fig. 6C). While oxidation on methionine (M100, M104, M426) was reduced in sample S3 with methionine added, the oxidation of tryptophan (W427) was not similarly affected (Fig. 6C).

Upon monitoring the glycosylation and deamidation on the N-terminal of GP41 subunit of HIV trimer, no chain truncation was observed. However, C-terminal peptide of GP41 subunit of HIV trimer with glycosylation sites showed minor loss of glycosylation and increased deamidation relative to the control in stressed samples S1 and S3 with respect to control and S2 (Fig. 6D). Similar to the trend of GP120, increased overall GP41 deamidation and oxidation were observed on stressed samples S1 and S3 in comparison to control and S2 (Fig. 6D, respectively).

Through detailed characterization of stability study batches and comparison with control, we obtained a list of attributes which includes sites of PTM modifications and peptide chain clipping termini to be monitored for the evaluation of stability characteristics of HIV trimer subunit vaccine. Further, we established an automated data processing workflow for peptide-based MAM analysis of HIV-1 trimers using the Byos software package by creating a workflow template (Figures S41-S42).

### Characterization of development-grade process representative and GMP manufactured vaccine using MAM

**Fig. 7:**
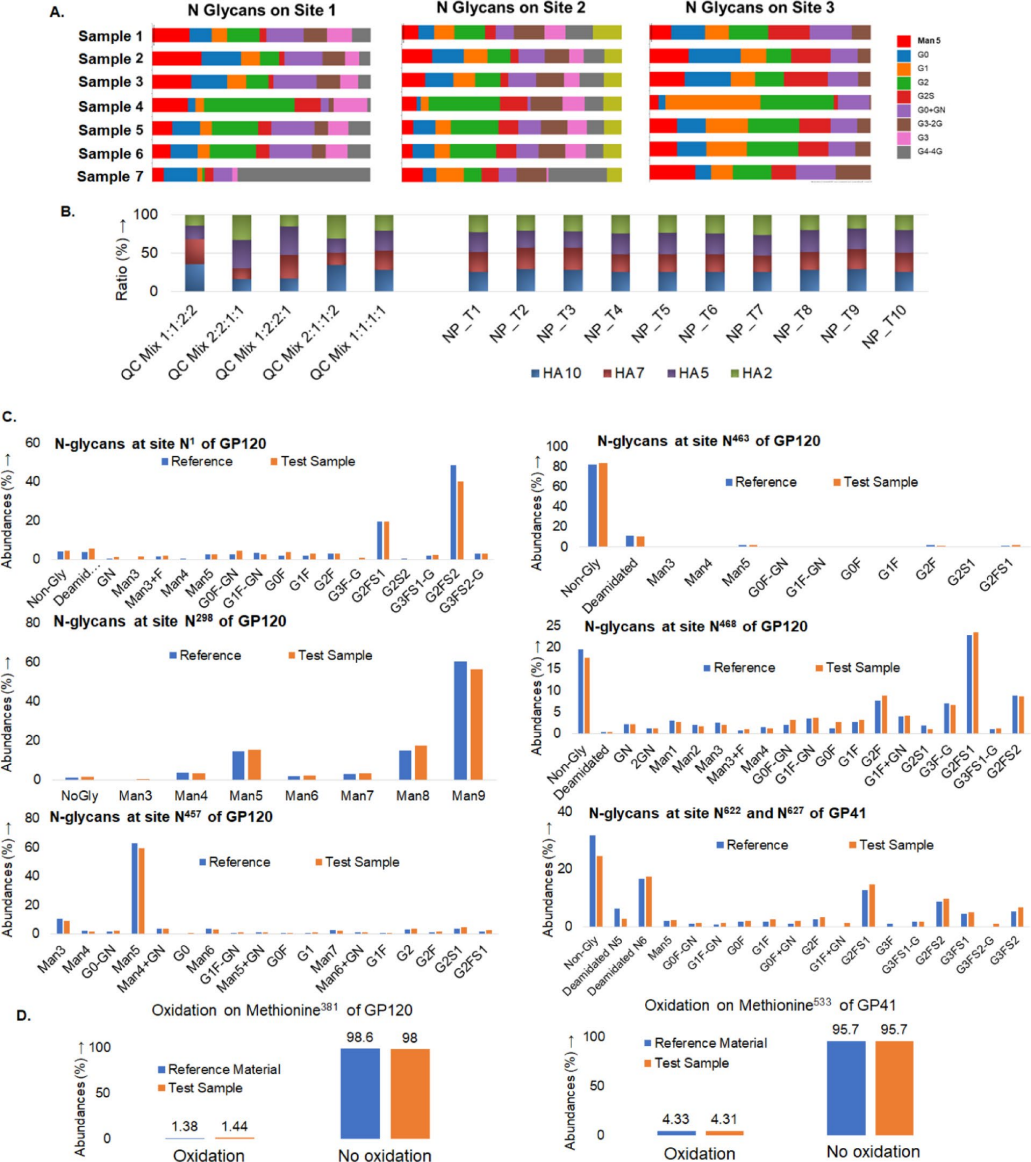
MAM for the characterization of vaccine test sample with reference (**A**) MAM stage 2 high throughput screening of glycosylation across HA10 samples using template created based on stage 1 characterization, (**B**) SteMos1 nanoparticle HA component ratio evaluation by MAM (QC—quality control, NP_T—nanoparticle test sample). Comparison of HIV trimer GP120 and GP41 subunits, (**C**) glycosylation and deamidation, (**D**) oxidation. This demonstrates the versatility of MAM in monitoring multiple critical quality attributes across different vaccine platforms and sample types, supporting both development and QC efforts.

Finally, the key PTM, HCP, and degradant attributes identified for both influenza stem vaccines and HIV envelope trimer during the various process development studies were evaluated for both development-grade process representative and cGMP manufactured vaccine samples. During this stage a ready-to-apply workflow template for the MAM was created in Byos software to monitor each protein component of vaccines. Different set of templates for the peptide mapping project creation with sequence, digestion enzyme, modification lists, and search filters were created for each of the four-protein components (HA2, HA5, HA7 and HA10) of influenza stem vaccines during different process development (Supp. Figure S41). Further, separate reporting template was also created for each protein component of influenza stem vaccines to monitor critical attributes at each process development step. During the stage 2 of MAM, the critical attributes were monitored during production using MAM parameters developed during stage 1. A high throughput stage 2 MAM based screening of glycosylation was conducted for the influenza HA10 stem components using project and reporting template created through detailed characterization stage (stage 1) (Fig. 7A, Supp Figure S42). To test the proof-of-concept of MAM for large sample sets, we evaluated an optimized MAM workflow by performing a test search on a batch of about a hundred process purification samples for the detection of HCPs and PTM change identifications (Supp. Figure S43). We have also employed MAM for the determination of ratio of each HA component (HA2, HA5, HA7 and H10) in the SteMos1 nanoparticle vaccine products (Fig. 7B). The ratio of each component was calculated by comparing the abundances of extracted ion chromatograms of peptides unique to each HA component (Supp. Table S2). The method was validated for repeatability and accuracy by using quality control samples with predetermined ratio of each HA component.

Similarly, the N-glycosylations and deamidation at critical sites on GP120 and GP41 subunits of HIV trimer vaccine test sample and reference were also tested after initiating a project creation and reporting template for MAM based on stage 1 characterization. Test samples and reference were similar (Fig. 7C). Similarly, the oxidation on HIV trimer were also found similar between the test sample and reference (Fig. 7D).

Stage 1 development of iMAM was conducted using MaxEnt1 deconvolution in MassLynx software. To establish a high-throughput screening process for vaccine subunits’ chain clipping via iMAM, an automated deconvolution and processing workflow was developed in the Byos intact mass analysis module. The deconvolution parameters for each set of deglycosylated protein subunits with varying masses were optimized. Based on these parameters, a project template for automated peak processing and quantification of chain cleavages in HIV trimers under heat stress conditions was created. This template was then evaluated for its applicability in determining chain cleavages of vaccine subunits by comparing heat-stressed samples with a reference control as part of stage 2 of iMAM (Supp. Figures S44-S45, Supp. Table S3).

## Discussion

This study demonstrates the first use of MAM to support the process development and characterization of heavily glycosylated protein vaccine molecules^6,12,21,22^. MAM is one of the most sensitive approaches with high throughput to characterize and quantitate site-specific glycosylation and other PTMs in biotherapeutics with a limited number of steps^9,11,13,16,23^. During Stage 1 of the MAM, an Influenza hemagglutinin tetravalent stem mosaic vaccine and its individual protein components, and an HIV envelope trimer protein subunit vaccine were characterized in detail, identifying key attributes altered during process optimization. We evaluated the N-glycosylation, extent of protein chain clipping, methionine modifications, deamidations, other PTMs, and host cell protein quantifications at each process development stage. The HA stem proteins from multiple Influenza HA strains (HA2, HA5, HA7, and HA10) expressed in different cell types (HEK293 and CHO cells) were assessed by MAM, and potential critical attributes were studied, quantified, and compared.

Comparison of all three glycosylation sites of each component of the tetravalent vaccine enabled the selection of clones that closely matched the same set of proteins expressed in HEK293 cells. Later, we employed MAM to understand how culture harvest conditions affect glycosylation and other PTMs on vaccine components. Our direct in-gel analysis method enabled high-throughput monitoring of culture conditions without tedious isolation or purification of the protein of interest. MAM determined changes in glycosylation in proteins isolated at different cell culture sampling days and, interestingly, identified unusual oxidation, dethiomethylation, and formylation on two methionines in the HA10 protein component of the influenza nanoparticle vaccine. MAM further helped identify the HCPs removed during each step of protein purification and assessed whether the purification process removed any particular type of PTM modification. Finally, MAM was employed to monitor test samples with appropriate reference molecules, and changes in the PTMs were tracked. MAM, in the form of intact subunit mass (iMAM) and peptide mapping, helped understand changes in protein integrity under different formulations and stability testing conditions. Disulfide shuffling analysis was not conducted on this study as proteins with high number of glycosylation necessitates additional protease digestions and deglycosylation to prevent interference of glycosylation in determining disulfide bonded peptide with and without reducing the protein.

An advantage of MAM is site-specific characterization of glycosylation. Glycosylation can also be analyzed by enzymatically releasing oligosaccharides thereby losing site-specific glycan heterogeneity information. Both intact mass-based and peptide mapping-based MAM have been developed to obtain site-specific glycosylation information on monoclonal antibodies^9,11^. While these methods work well for monoclonal antibodies or proteins with fewer glycosylation sites, applying them to understand glycan diversity on vaccines is challenging due to the heavy glycan occupancy at multiple sites on the protein backbone^23^. Our approach addresses a critical gap in current analytical capabilities by handling the complexity of subunit protein vaccines. Enhanced precision and efficiency in glycosylation site characterization are achieved, supporting the development of vaccines through comprehensive insights into their structural attributes. By extending the application of MAM to more complex biotherapeutics, this work paves the way for broader adoption of this analytical approach in vaccine development and quality control.

To effectively characterize PTMs at numerous sites while eliminating false detections, we devised a strategy to segregate glycosylation sites and PTMs into different groups based on the protein component they belong to or were found critical in prior studies. Each modified peptide was thoroughly investigated manually for retention time alignment, MS1 precursor isotopic pattern, MS2 fragmentation, and peak picking. During HCP searches, we identified that glycosylated peptides of vaccine protein components were often picked up as false HCPs due to low-quality fragmentation of peptide backbones observed in glycopeptides. These were manually identified and filtered out before the quantification process. We verified that the annotated sialylated and non-sialylated glycans have different retention times as number of sialylations causes the peptides to migrate to higher retention times^24^. We ensured that glycopeptides preferably had the Y1 fragment (peptide + GlcNAc) or higher intensity b or y ion peptide fragments along with signature glycan fragments to confirm as true detection^25^. For deamidated and oxidized peptides, we ensured the MS1, MS2, and retention times to eliminate any false assignment due to in-source modification^26,27^.

Another observation was that Byos software did not pick up peptides and glycopeptides lacking MSMS fragmentation spectra. In such cases, the ‘create new from current’ feature of Byos software was used to pick glycopeptide peaks based on MS1 spectra. For peptides with unusual modifications on two methionines separated by only four residues, we manually verified that oxidation, dethiomethylation, and formylation modifications occurred only on the methionines. For a tryptic peptide of HA10 with two methionine and one glycosylation site, we observed close to 300 peptide spectrum matches (PSM) due to about 33 unique glycans and combinations of modifications on two methionines along with a minor fraction of asparagine deamidation. Dethiomethylation (− 48 Da) is a rare modification resulting from the elimination of the methionine side chain post-oxidation^28^. Formylation was another rare modification observed on these two methionines of HA10^29^. It is intriguing that such modifications occurred in about 20–30% of M42 and M47 of HA10, while methionines at other sites showed only marginal levels of oxidation. Other HA components of the SteMos1 influenza vaccine, such as HA2, HA5, and HA7, have only a single methionine at this homologous site, and no excessive methionine modification was observed. We are investigating the possible reasons for such selective methionine modifications and whether they have implications in the tetravalent nanoparticle’s integrity, stability, or aggregation. We incorporated all the above-mentioned manual curation criteria during the stage 1 MAM which comprises detailed understanding of the critical parameters for each protein subunit. Further, an automated data processing template was created for each subunit vaccine HA component as part of the execution of stage 2 of MAM. This approach of segregating glycosylation sites and post-translational modifications (PTMs) based on protein components or critical findings from prior studies enhances the accuracy of characterization and eliminates false detections. Despite the advancements, the method still necessitates laborious manual data processing, including thorough investigation of modified peptides for retention time alignment, MS1 precursor isotopic patterns, MS2 fragmentation, and peak picking. This meticulous manual curation is essential to ensure the reliability of the data and to filter out false identifications, particularly in the context of host cell protein (HCP) searches and glycopeptide analysis. The ongoing need for such detailed manual processing underscores the complexity of mass spectrometry peptide mapping and the importance of precise analytical techniques in biopharmaceutical development. We also evaluated the precision in the sample preparation, data acquisition and data processing by performing triplicates of peptide mapping experiments on HA10 component and observed that the standard deviations are under 1.4.

The application of MAM enabled detailed profiling of post-translational modifications (PTMs), which played a critical role in guiding cell line and clone selection during vaccine development. Glycosylation screening across multiple expression systems—including HEK293 and CHO cells—revealed significant differences in site occupancy, glycan heterogeneity, and structural fidelity. Clones derived from CHO cells often exhibited altered glycan profiles, including increased high-mannose content and reduced sialylation, compared to the original HEK-derived clones. By comparing PTM profiles across sub-clones, we identified CHO clones that closely matched the glycosylation patterns of the original HEK clones, ensuring consistency in antigen presentation and immunogenicity. Additionally, MAM-based monitoring of oxidation levels across different culture conditions provided insights into oxidative stress and protein stability. Clones cultured under suboptimal conditions showed elevated methionine oxidation, prompting adjustments in media composition and process parameters. These findings underscore the utility of MAM not only in structural characterization but also as a decision-making tool for selecting optimal cell lines and clones that produce vaccine candidates with desirable PTM profiles.

Key proof-of-concepts demonstrated in this study include the definition of criteria for potential critical attributes and development of a workflow adaptable for executing stage 2 MAM for high-throughput screening of subunit vaccine molecules at different manufacturing processes and final product characterization. For the future, we are developing an automated sample preparation workflow in order to increase the throughput of MAM to thousands of samples from different process optimization conditions. Significant challenges will include addressing high data volumes through coordinating the data acquisition, data transfer, data processing, mitigation of false identification and compiling the data for a meaningful interpretation^30^. Additionally, we demonstrated that combining iMAM and MAM was an effective tool for monitoring potential degradation of the HIV trimer under high-heat stress conditions. Automation for the iMAM-based determination of HIV trimer cleavages through software tools was also effective. This study demonstrates the effective application and successful integration of MAM in the product and process development of highly complex and challenging protein vaccines, exemplified by the Influenza hemagglutinin stem mosaic and HIV envelope trimer vaccines.

Vaccine candidates included in this study were selected based on their progression through early-stage development pipelines, with emphasis on candidates undergoing cell line and clone selection, upstream process optimization, and stability evaluations. Priority was given to molecules with known glycosylation complexity and relevance to seasonal or pandemic preparedness programs (e.g., influenza and HIV subunit vaccines). For data analysis, measures were taken to assess the reproducibility and variability across replicates and process conditions. We evaluated consistency of critical quality attributes (CQAs) observed such as glycosylation site occupancy, intact mass, and PTM profiles across the tested molecules and samples. These analyses supported the identification of robust markers for product quality and facilitated the development of high-throughput MAM workflows suitable for process monitoring.

Applying MAM to glycosylated subunit vaccine candidates with high glycan heterogeneity presented several technical and analytical challenges. One major difficulty was the exponential increase in data complexity due to multiple glycosylation sites and diverse glycan structures, which complicated site-specific glycan assignment and quantitation. To address this, we implemented a data processing workflow that addressed the glycosylation site analysis after defining whether they are a critical attribute for the functional efficacy of the vaccine or not, allowing for iterative refinement of selected glycan site mapping. We also encountered limitations in software compatibility and automation, which were mitigated by integrating multiple data analysis platforms and by choosing a data processing platform which can handle complex PTM heterogeneity. Additionally, variability in sample preparation and digestion efficiency was minimized through standardized protocols and internal controls. These strategies collectively enabled reliable monitoring of glycosylation and other critical attributes across diverse vaccine candidates.

To enable MAM as a QC and release assay for glycosylated subunit vaccines, several foundational steps must be established. Robust method qualification and validation protocols to establish system suitability criteria, acceptance ranges for critical quality attributes (CQAs), and ensuring reproducibility are required for QC application of MAM. Regulatory alignment is critical in MAM workflows to ensure guidelines for biologics, including documentation of method performance, data integrity, and traceability are followed. The software tools for data processing and attribute reporting must be validated and integrated into QC systems, with clear audit trails and compliance features.

## Supplementary Information

Below is the link to the electronic supplementary material.

Supplementary Material 1

Supplementary Material 2

Supplementary Material 3

## Data Availability

The datasets generated and/or analyzed during the current study are available in the GlycoPOST repository. Raw data access: https://glycopost.glycosmos.org/preview/188325300068202b03cb324, Pin: 6697.
