# Supplementary material for: Multi-attribute monitoring (MAM) methodology for glycosylated subunit vaccines
Source: Sci Rep. 2025 Nov 21;15:41198. doi: 10.1038/s41598-025-24922-8 (PMC12638818; doi:10.1038/s41598-025-24922-8)
Supplement: Supplementary file 1 — Supplementary Material 1 [file 41598_2025_24922_MOESM1_ESM.docx]

**Supporting information**

**Multi-Attribute Monitoring (MAM) Methodology for Glycosylated Subunit Vaccines**

Asif Shajahan^1^, Lisa M. Jenkins^2^, Nathan Barefoot^1^, Darielys Maldonado^1^, Jeremy Wolff^1^, Yanhong Yang^1^, Lisa A. Kueltzo^1^, Valerie Ficca^1^, Elizabeth Scheideman^1^, Ivan Loukinov^1^, Carl Carruthers^1^, Dorra Benmohamed^1^, Daniel B. Gowetski^1^, Rong Jiang^1^, Sylvie R. Yang^1^, Kevin Carlton^1^, Jason D. Gall^1^, Q. Paula Lei^1^

^1^Vaccine Production Program Laboratory, Vaccine Research Center, National Institute of Allergy and Infectious Diseases, National Institutes of Health, Gaithersburg, MD, USA

^2^Laboratory of Cell Biology, Center for Cancer Research, National Cancer Institute, National Institutes of Health, Bethesda, MD, USA

Table S1: SteMos components clones tested by MAM

| SteMos Component | HA2 | HA5 | HA7 | HA10 |
| --- | --- | --- | --- | --- |
| Clones compared by MAM | Clone A | Clone A | Clone A | Clone A |
|  | Clone B | Clone B | Clone B | Clone B |
|  | Clone C | Clone C | Clone C | Clone C |
|  |  |  |  | Clone A1 |
|  |  |  |  | Clone B1 |
|  |  |  |  | Clone B2 |
|  |  |  |  | Clone C1 |
|  |  |  |  | Clone C2 |
|  |  |  |  | Clone C3 |

Figure S1: Relative abundances of N-glycans on Site 1 (**DQICIGYHAN^10^N^11^STEK)** of HA2

Figure S2: Relative abundances of N-glycans on Site 2 (**N^23^VTVTHAK**) of HA2

Figure S3: Relative abundances of N-glycans on Site 3 (**N^178^GTYDYPK**) of HA2

HA2 Clone A

HA2 Clone B

HA2 Clone C

Figure S4: Asparagine deamidation on peptide ELGN^159^GCFEFYHK of HA2

HA2 Clone A

HA2 Clone B

HA2 Clone C

Figure S5: Asparagine deamidation on peptide LGN^247^AYYK of HA2

HA2 Clone A

HA2 Clone B

HA2 Clone C

Figure S6: Methionine oxidation on peptide AFDGITN^102^MVNSVIEK of HA2

HA2 Clone A

HA2 Clone B

HA2 Clone C

Figure S7: Methionine oxidation on peptide M^110^GSGGSGTYNAELLVL of HA2

Figure S8: Relative abundances of N-glycans on Site 1 (DQICIGYHAN^10^N^11^STEQVDTIMEK**)** of HA5.

Figure S9: Relative abundances of N-glycans on Site 2 (N^23^VTVTHAQDIGSGLVLATGLR**)** of HA5.

Figure S10: Relative abundances of N-glycans on Site 3 (**N^178^GTYNYPQYSEEAR)** of HA5.

Figure S11: Asparagine deamidation on peptide CDN^170^ECMESIR of HA5.

HA5 Clone A

HA5 Clone B

HA5 Clone C

Figure S12: Asparagine deamidation on peptide ELGN^159^GCFEFYHK of HA5.

HA5 Clone A

HA5 Clone B

HA5 Clone C

Figure S13: Asparagine deamidation on peptide ALELDP**N^271^N^272^**AEAWY**N^278^**LG**N^281^**AYYER of HA5.

HA5 Clone A

HA5 Clone B

HA5 Clone C

Figure S14: Asparagine deamidation on peptide LDPN^305^N^306^ADAMQN^312^LLN^315^AKof HA5.

HA5 Clone A

HA5 Clone B

HA5 Clone C

Figure S15: Methionine oxidation on peptide CDNECM^173^ESIR of HA5.

HA5 Clone A

HA5 Clone B

HA5 Clone C

Figure S16: Methionine oxidation on peptide LDPNNADA**M^310^**QNLLNAK of HA5.

Figure S17: Relative abundances of N-glycans on Site 1 (DKICLGHHAVSN^12^GTK**)** of HA7.

Figure S18: Relative abundances of N-glycans on Site 2 (GVEVVN^28^ATELVFPGCGVLK**)** of HA7.

Figure S19: Relative abundances of N-glycans on Site 3 (CDDDCMASIRN^183^NTYDHSK**)** of HA7.

HA7 Clone A

HA7 Clone B

HA7 Clone C

Figure S20: Asparagine deamidation on peptide ALELDPN^276^N^277^AEAWYN^283^LGN^286^AYYER of HA7.

HA7 Clone A

HA7 Clone B

HA7 Clone C

Figure S21: Asparagine deamidation on peptide LDPN^310^N^311^ADAMQN^317^LLN^320^AK of HA7.

HA7 Clone A

HA7 Clone B

HA7 Clone C

Figure S22: Methionine oxidation on peptide LDPNNADAM^315^QNLLNAK of HA7.

HA7 Clone A

HA7 Clone B

HA7 Clone C

Figure S23: Methionine oxidation on peptide CDDDCM^178^ASIR of HA7.


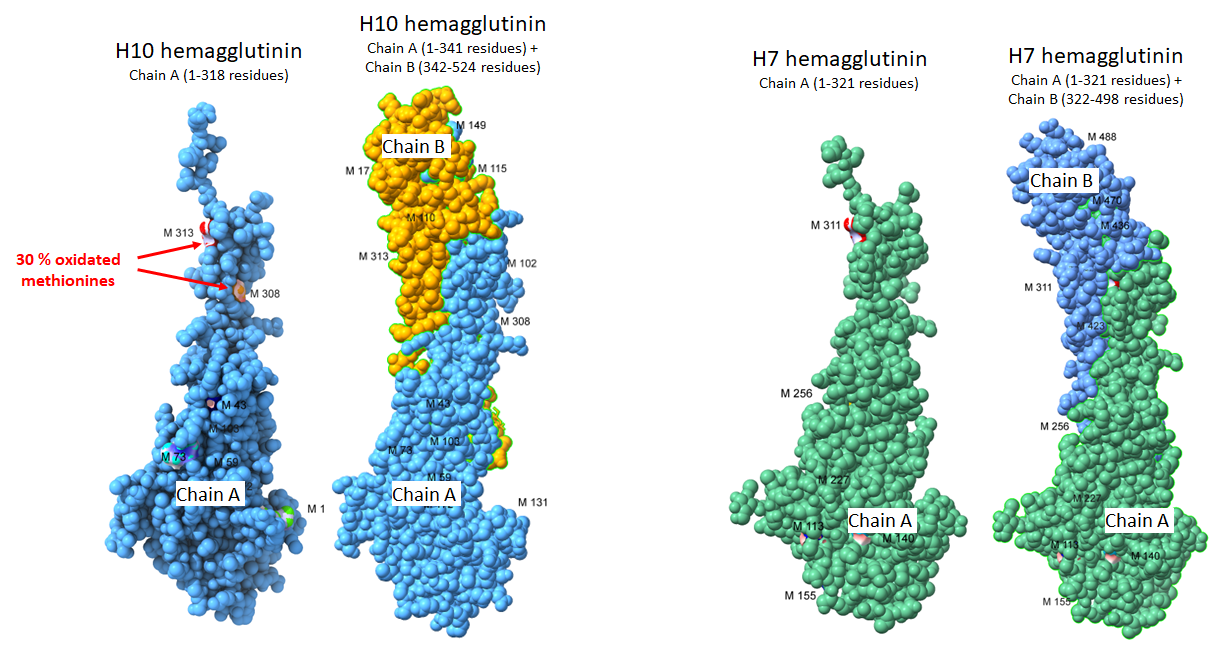
Figure S24: Comparison of excessively oxidized methionine sites M308 and M313 of full-length hemagglutinin from H10 influenza strain (PDB ID: 4WSX) with methionine of full-length hemagglutinin from H7 influenza strain (PDB ID: R4NN21). Methionine numbering as based on H10 and H7 PBD files.


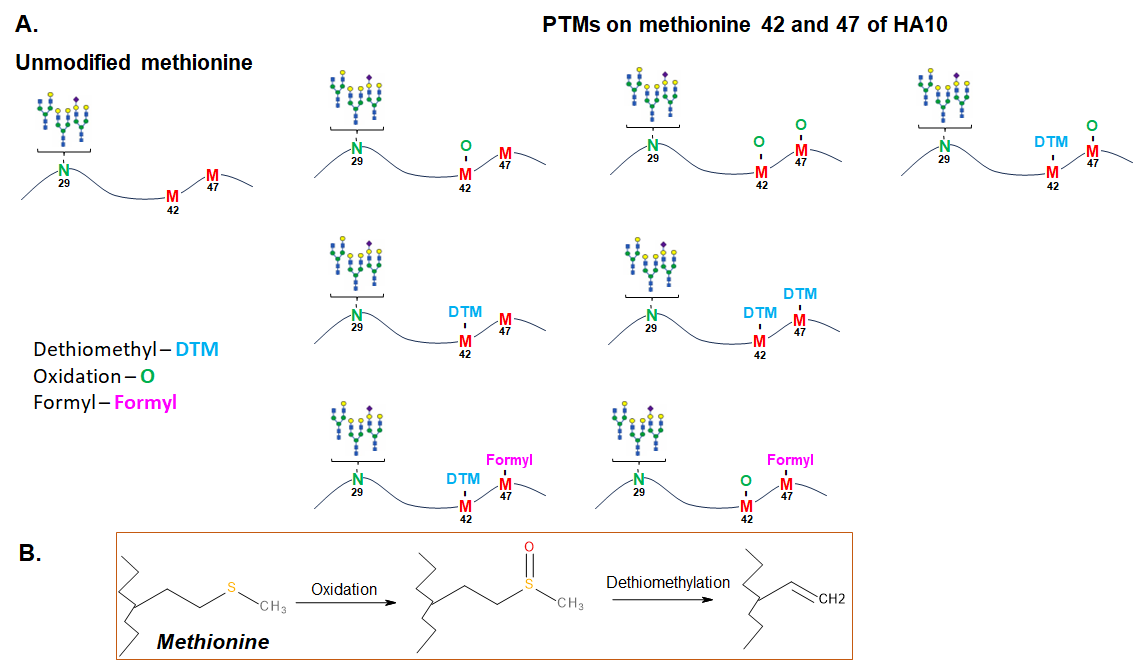


Figure S25: A. Oxidation and other PTM on methionines 42 and 47 of HA10, B. Reaction scheme showing methionine oxidation leading to dethiomethylation.


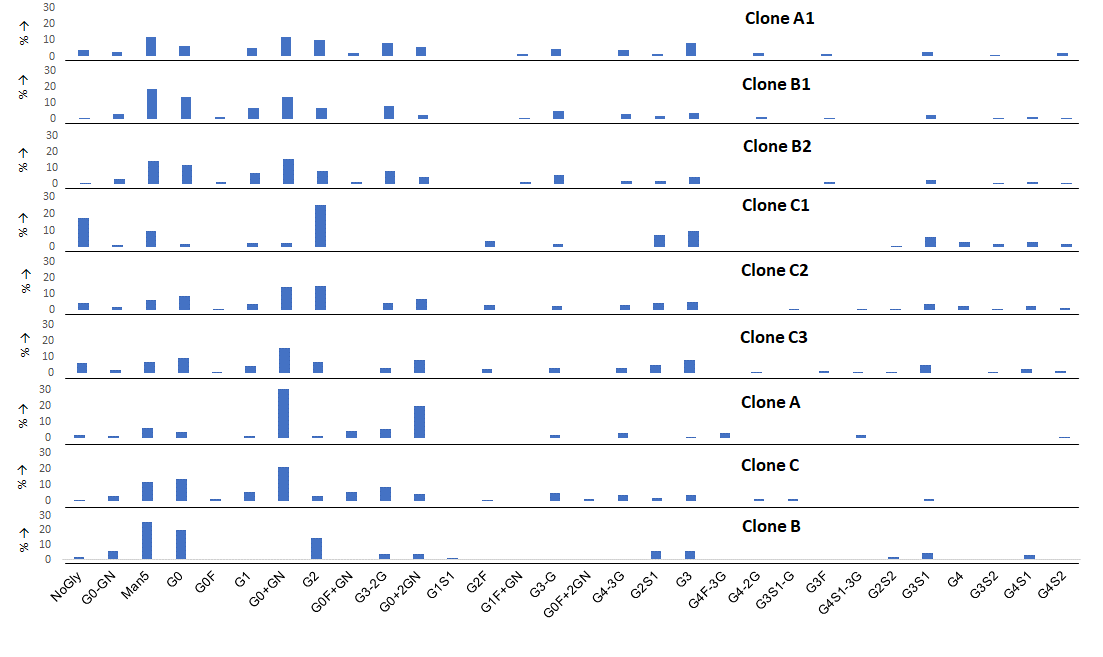


Figure S26: Relative abundances of N-glycans on Site 2 (TLTNEQEEVTNATELVFPGCGVL) of HA10


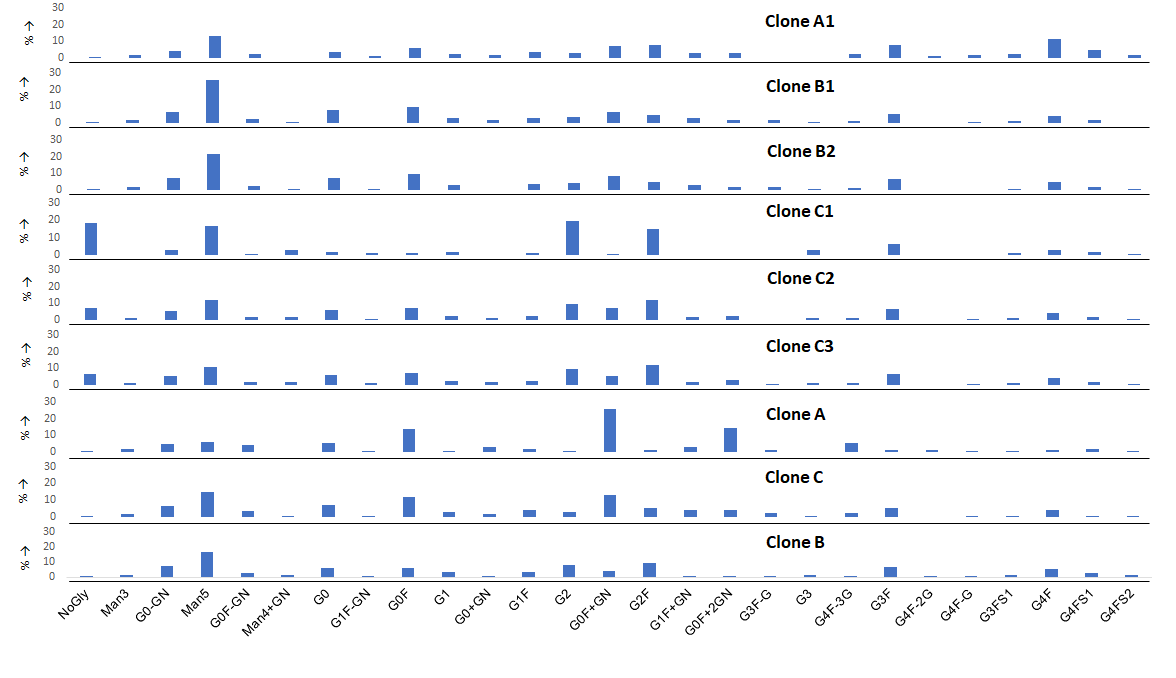


Figure S27: Relative abundances of N-glycans on Site 3 (NNTYDHSQYR) of HA10


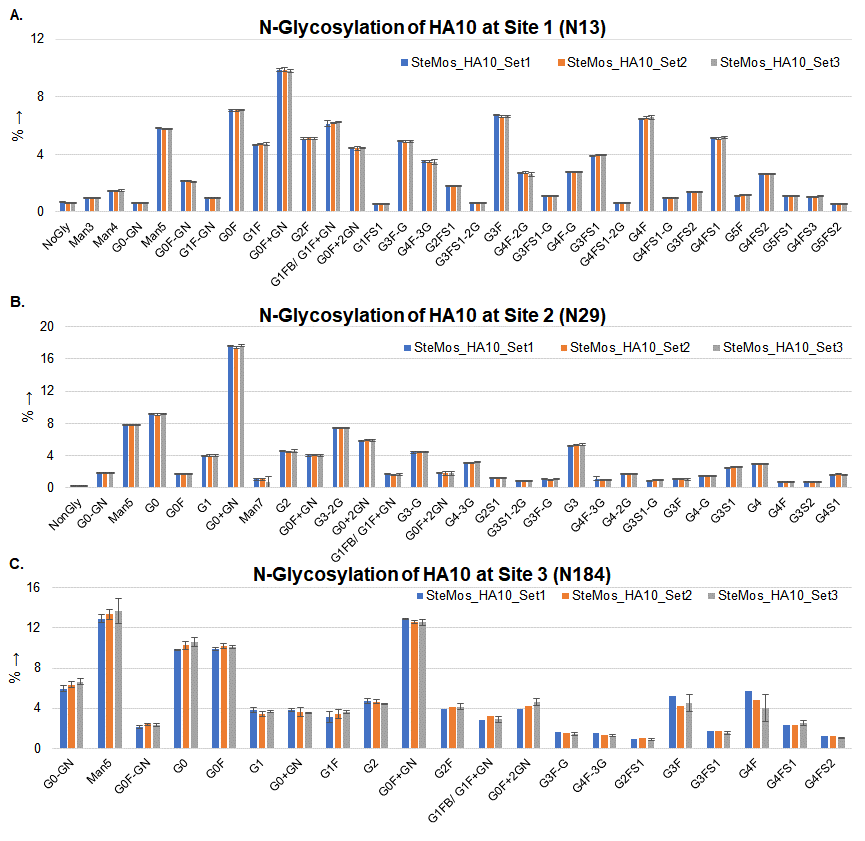


Figure S28: Comparison of sample preparation replicates (Sets 1, 2, 3) and LC-MS runs (Runs 1, 2, 3) for the evaluation of sample preparation and data acquisition variability; relative abundances of N-glycans on A. Site 1, B. Site 2 and C. Site 3 of SteMos HA10.


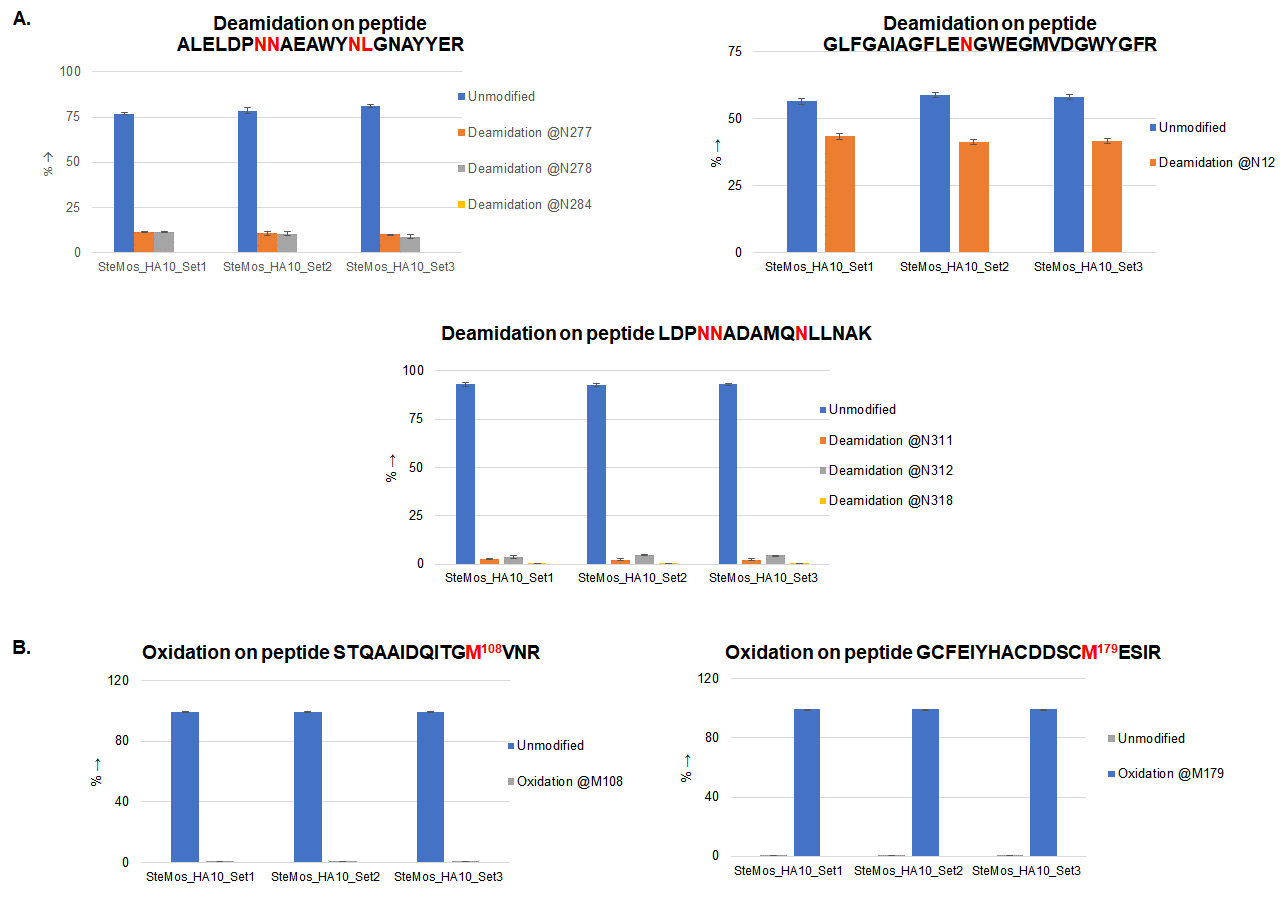


Figure S29: Comparison of sample preparation replicates (Sets 1, 2, 3) and LC-MS runs (Runs 1, 2, 3) for the evaluation of sample preparation and data acquisition variability; relative abundances of A. deamidation and B. oxidation on SteMos HA10.


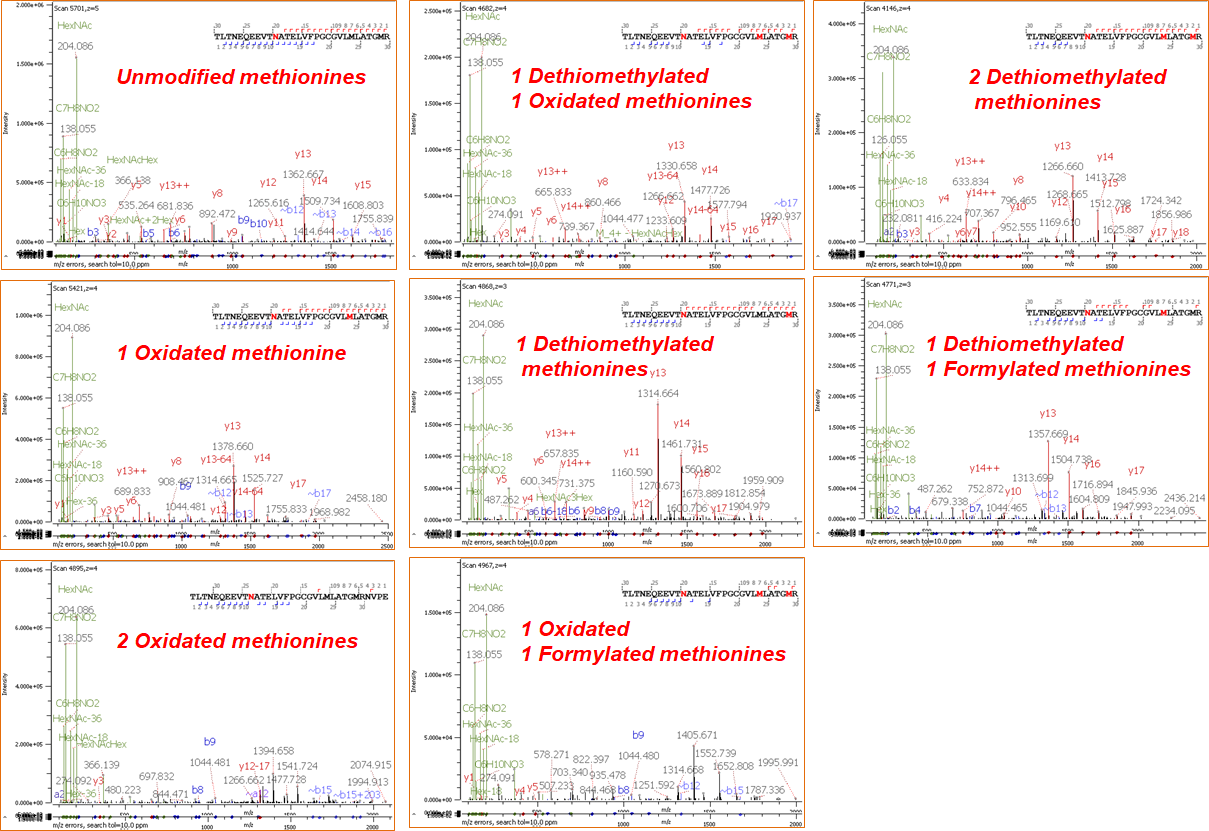


Figure S30: Representative Byos annotated MS/MS spectra of unmodified and methionine oxidated sites M42 and M47 of peptide TLTNEQEEVTNATELVFPGCGVLM^42^LATGM^47^R from HA10. y13 ion showing the mass shift based on the modifications on methionines.


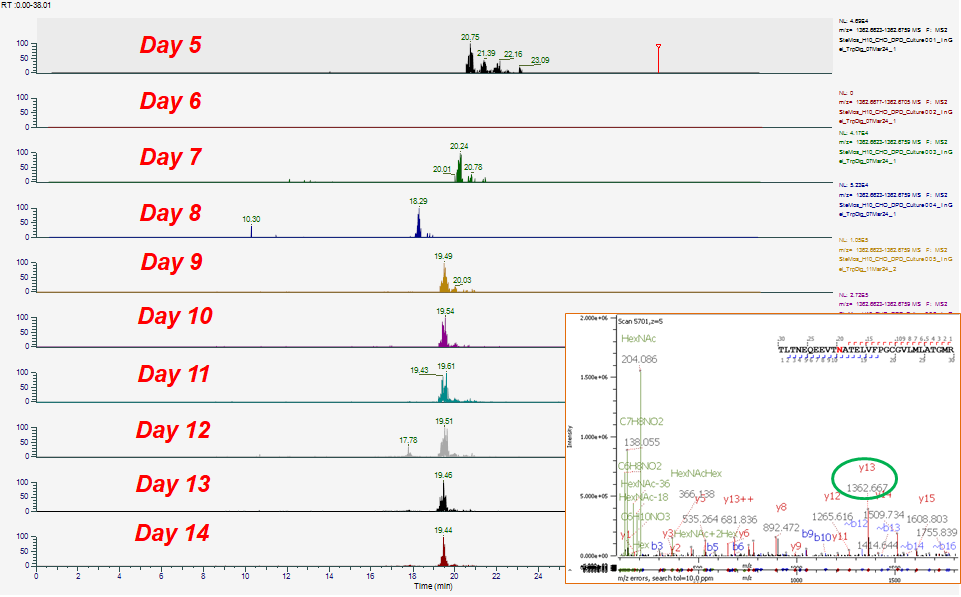


Figure S31: Unmodified HA10 peptide TLTNEQEEVTNATELVFPGCGVLM^42^LATGM^47^R across different days of harvest - XIC of 1362.6691 (y13) fragment.


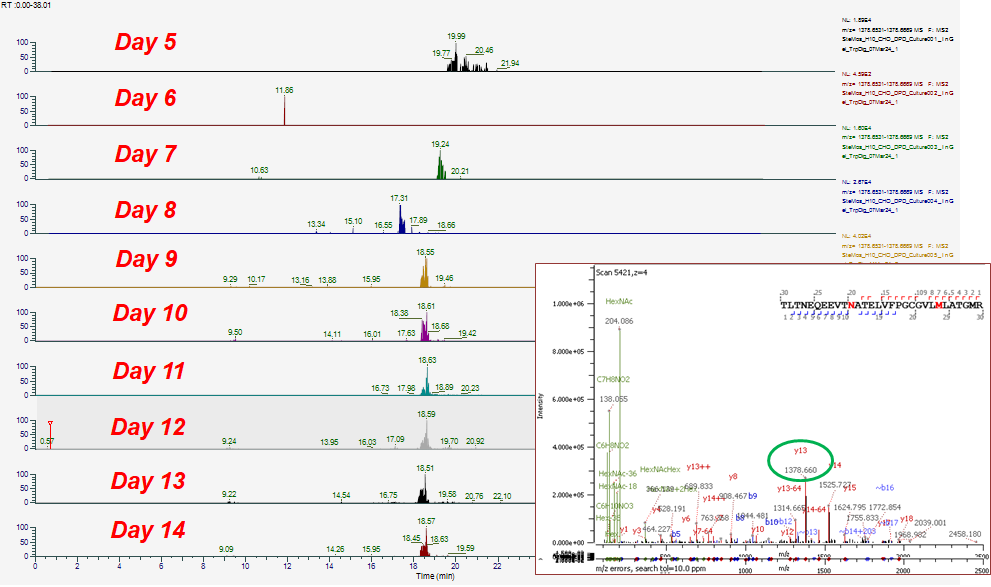


Figure S32: One methionine oxidation on HA10 peptide TLTNEQEEVTNATELVFPGCGVLM^42^LATGM^47^R across different days of harvest - XIC of 1378.660 (y13) fragment.


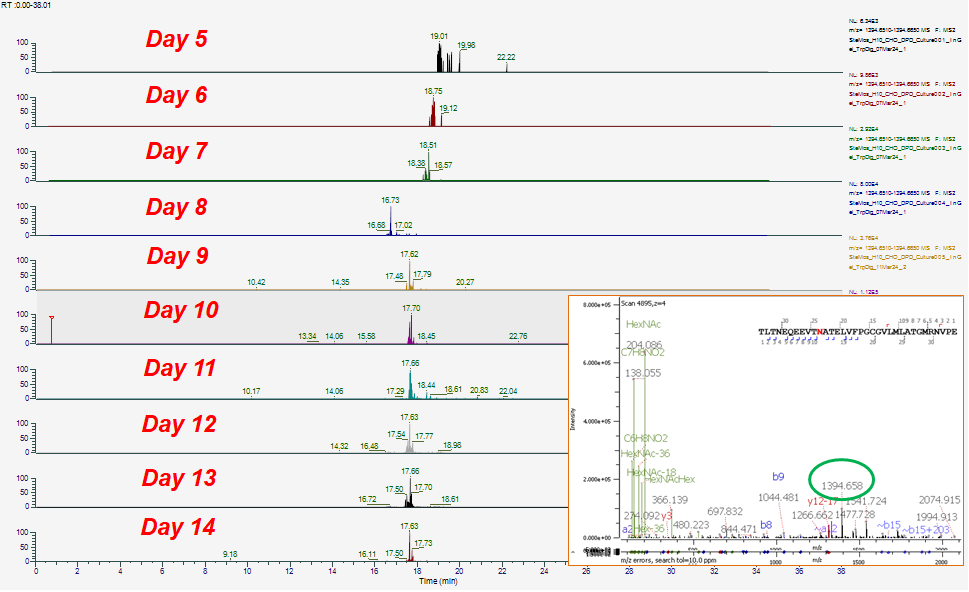


Figure S33: Two methionine oxidation on HA10 peptide TLTNEQEEVTNATELVFPGCGVLM^42^LATGM^47^R across different days of harvest - XIC of 1394.658 (y13) fragment.


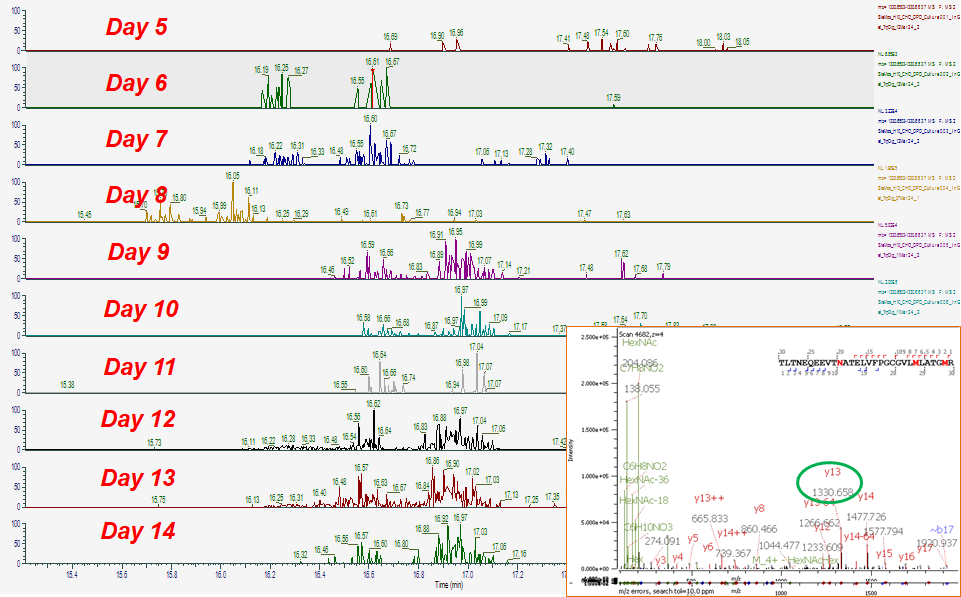


Figure S34: One methionine oxidation and one dethiomethylation on HA10 peptide TLTNEQEEVTNATELVFPGCGVLM^42^LATGM^47^R across different days of harvest - XIC of 1330.6570 (y13) fragment.


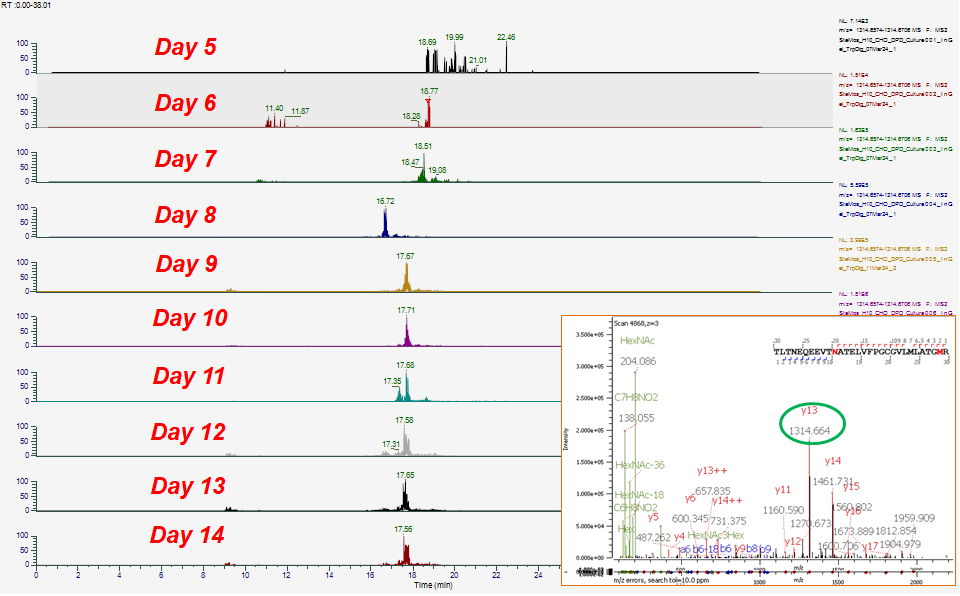


Figure S35: One methionine dethiomethylation on HA10 peptide TLTNEQEEVTNATELVFPGCGVLM^42^LATGM^47^R across different days of harvest - XIC of 1314.6640 (y13) fragment.


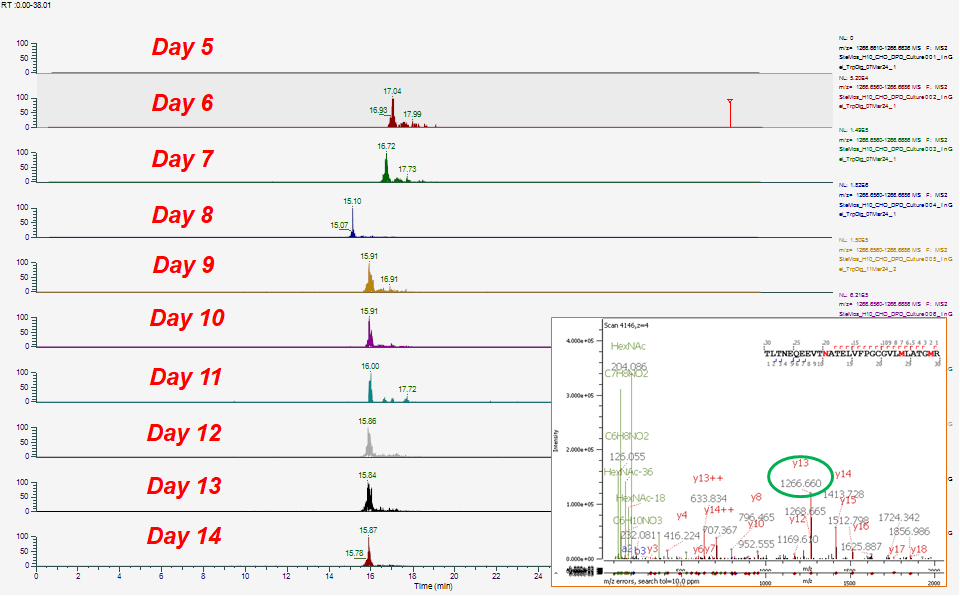


Figure S36: Two methionine dethiomethylation on HA10 peptide TLTNEQEEVTNATELVFPGCGVLM^42^LATGM^47^R across different days of harvest - XIC of 1266.6623 (y13) fragment.


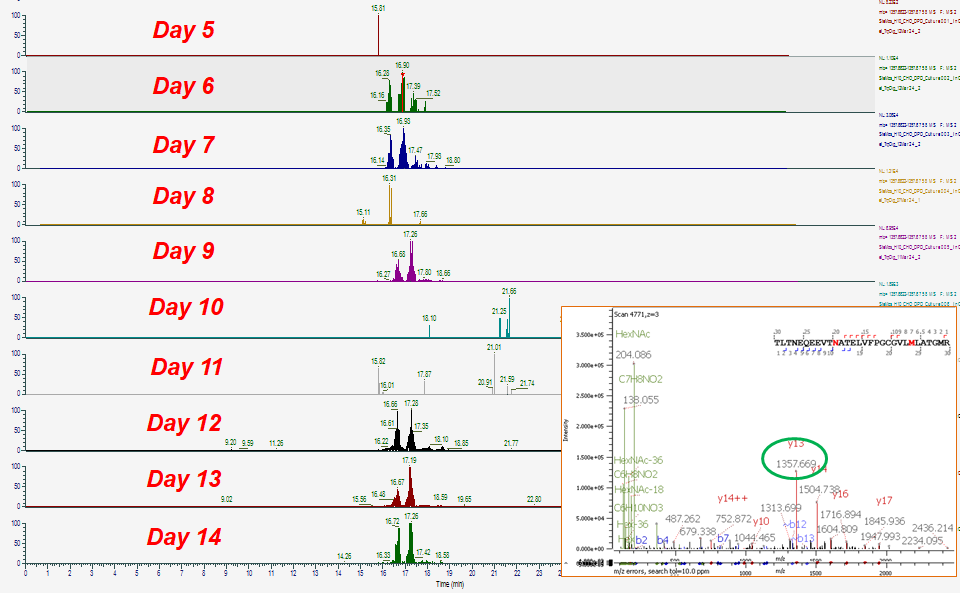


Figure S37: One dethiomethylation and one formylation modification on HA10 peptide TLTNEQEEVTNATELVFPGCGVLM^42^LATGM^47^R across different days of harvest - XIC of 1357.6690 (y13) fragment.


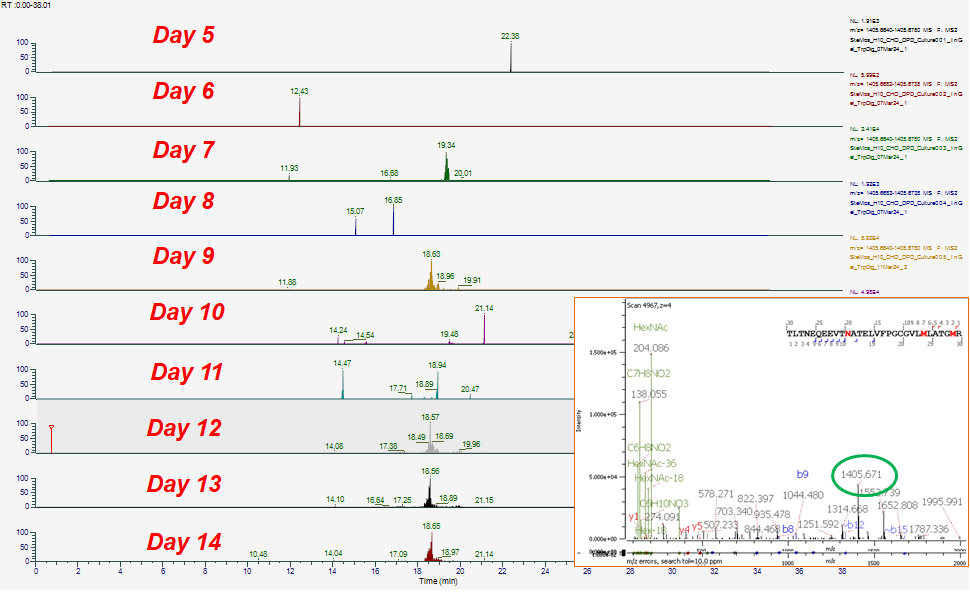


Figure S38: One methionine oxidation and one formylation on HA10 peptide TLTNEQEEVTNATELVFPGCGVLM^42^LATGM^47^R across different days of harvest - XIC of 1405.671 (y13) fragment.


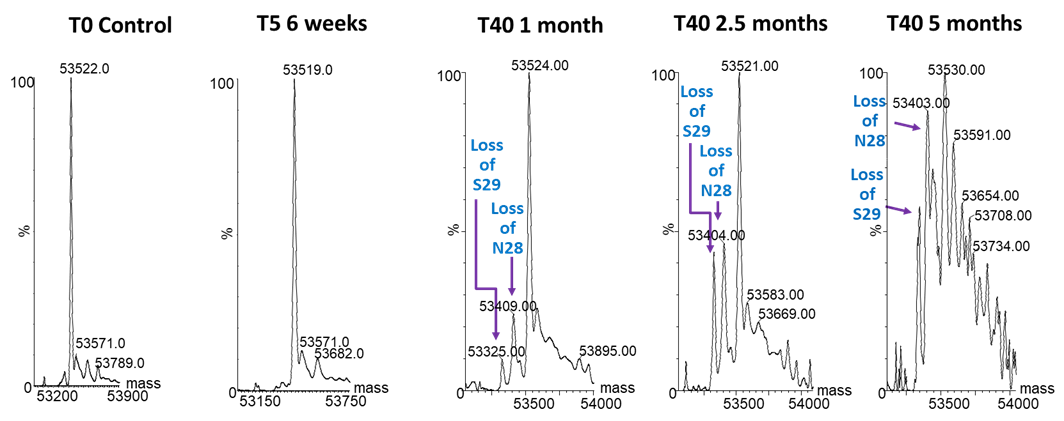


Figure S39: N-Chain cleavage observed on GP120 during elevated temperature treatment for different time periods**.**


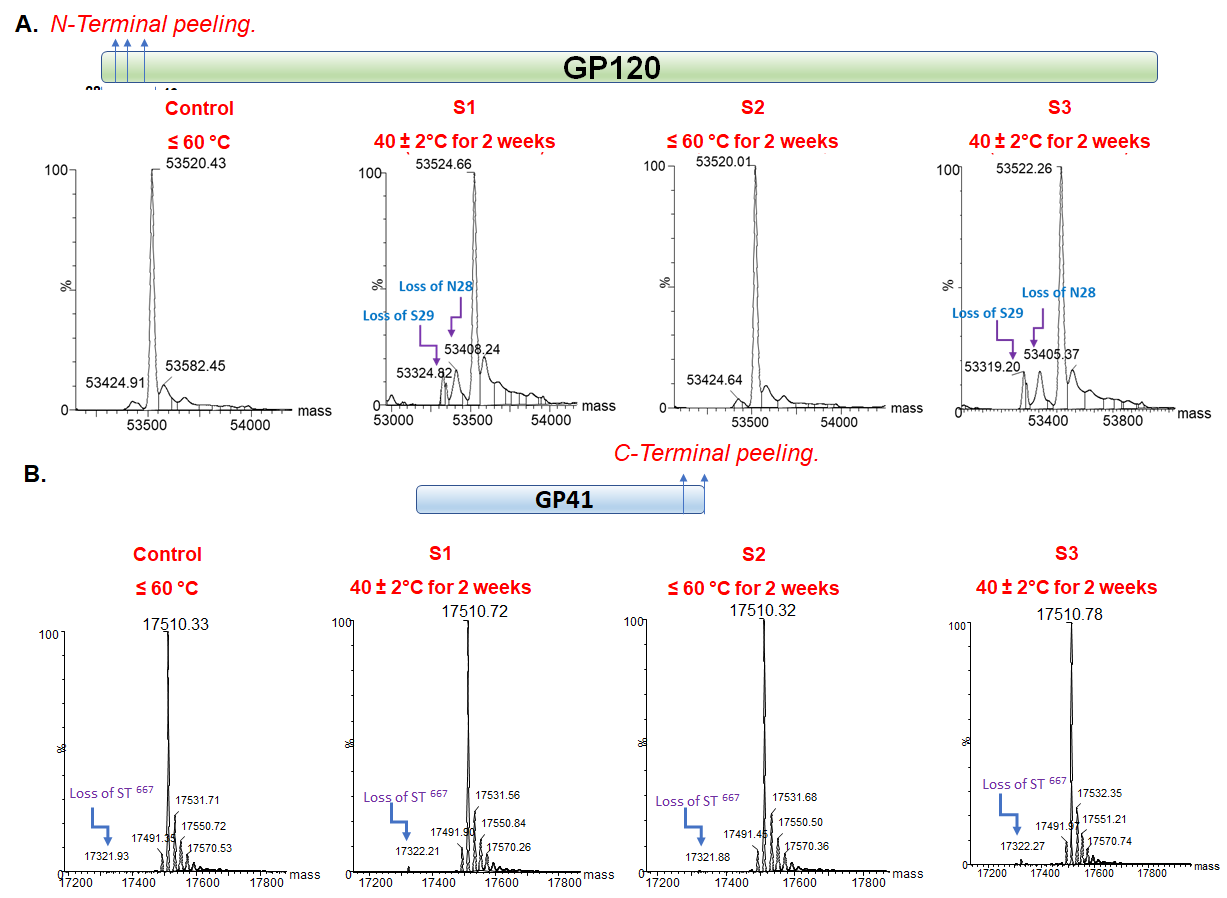


Figure S40: MAM by subunit MS analysis for chain clipping determination on deglycosylated samples in PBS, A. GP120 N-terminal cleavage, B. GP41 C-terminal cleavage.


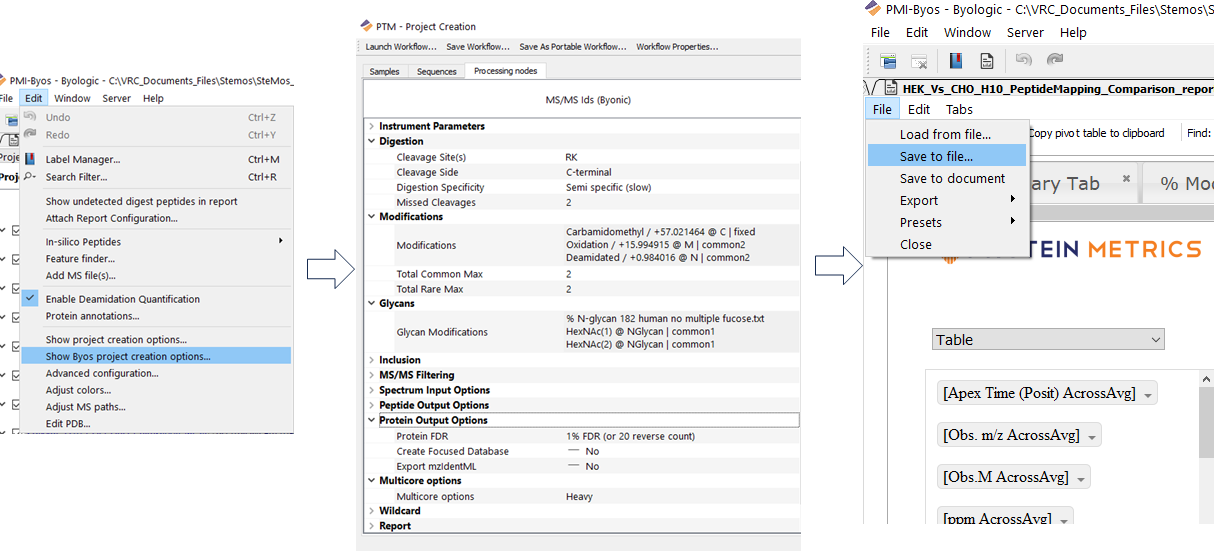


Figure S41: Creating Byos search template based on detailed stage 1 analysis and PTM characterization. Similar set of templates were created for each vaccine development process.


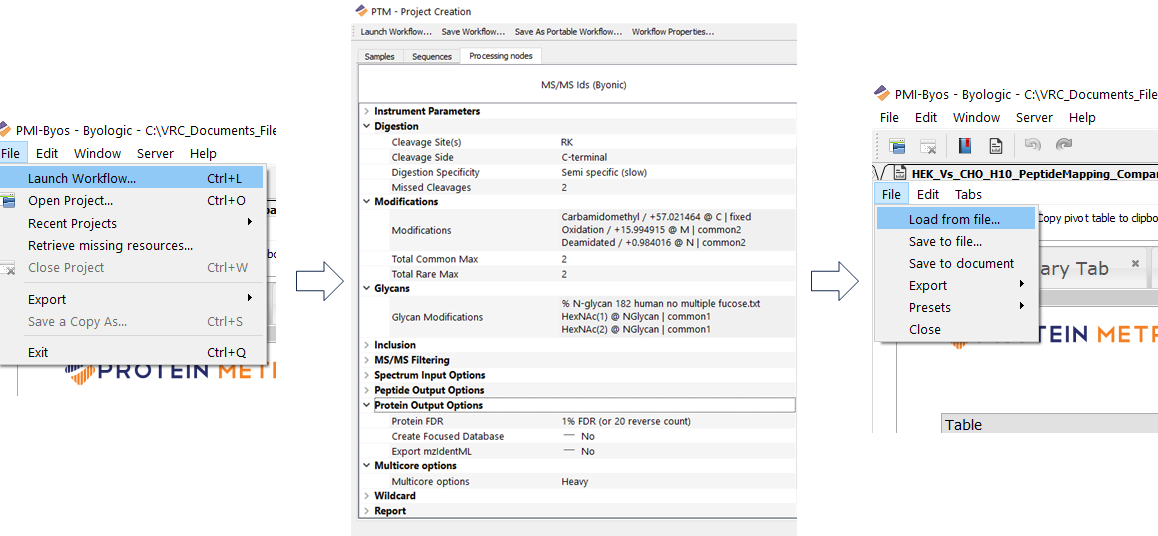


Figure S42: Performing MAM search on Byos software (stage 2) based on template created from stage 1 search.


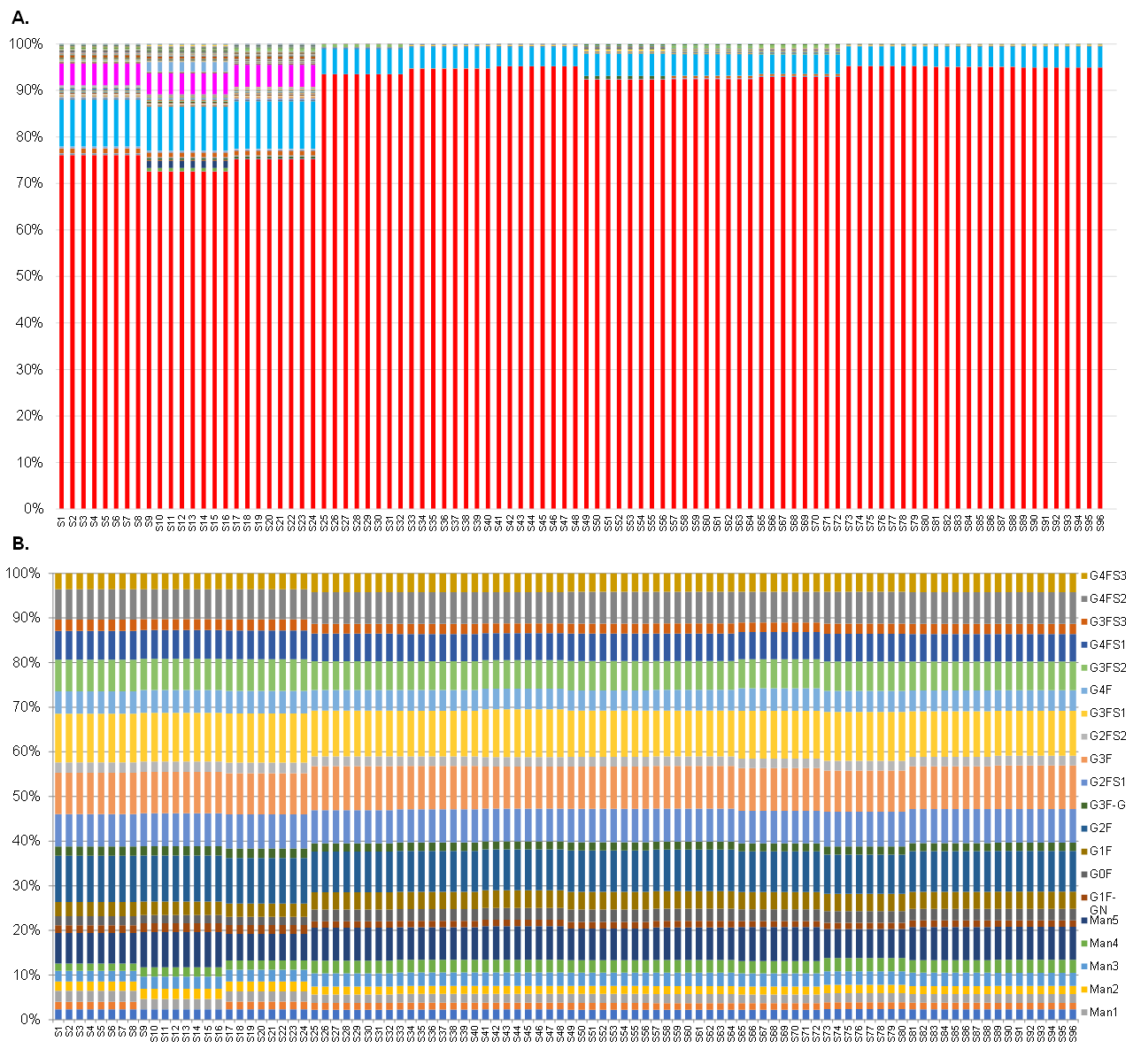


Figure S43: MAM high throughput test search (96 samples) output; A. HCPs detected on SteMos HA7 protein component of Influenza nanoparticle vaccine. B. N-glycosylation profile at site 1 of SteMos HA7 protein component of Influenza nanoparticle vaccine.


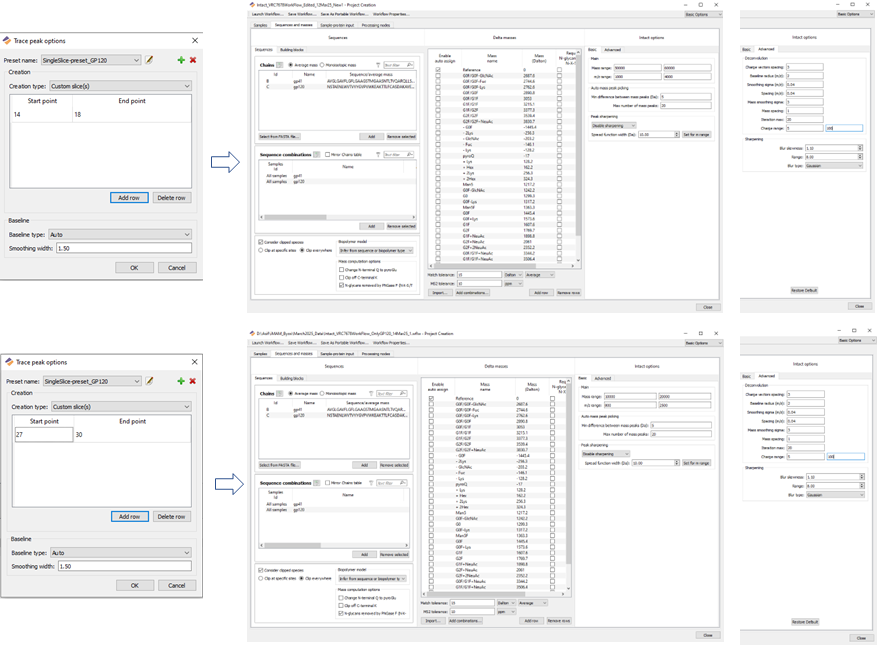


Figure S44: Performing iMAM search on Byos software (stage 2) based on template created from stage 1 optimization.

| 1. **QC Samples: SteMos_Components Mix Ratio** | | | | | | | | | | | | | | | |  |  |  |  |
| --- | --- | --- | --- | --- | --- | --- | --- | --- | --- | --- | --- | --- | --- | --- | --- | --- | --- | --- | --- |
| **HA Component Name** | **Unique Peptides** | **Retention Time (min)** | **Calc.M** | **Charge States** | **1_1_1_1** | | **1_1_2_2** | | **2_2_1_1** | | | **1_2_2_1** | | **2_1_1_2** | |  |  |  |  |
| HA2 | AFDGITNMVNSVIEK | 17.1 | 1636.81 | 2,3 | 20.4 | | 14.3 | | 32.9 | | | 14.9 | | 30.7 | |  |  |  |  |
| HA5 | AIDGVTNMVNSIIDK | 16.5 | 1588.81 | 2,3 | 25.9 | | 17.7 | | 36.5 | | | 37.0 | | 18.9 | |  |  |  |  |
| HA7 | STQSAIDQITGMVNR | 14.4 | 1619.79 | 2,3 | 25.0 | | 32.0 | | 13.9 | | | 31.0 | | 15.2 | |  |  |  |  |
| HA10 | STQAAIDQITGMVNR | 14.0 | 1603.8 | 2,3 | 28.6 | | 36.0 | | 16.7 | | | 17.1 | | 35.2 | |  |  |  |  |
| 1. **SteMos Nanoparticle Test Samples** | | | | | | | | | | | | | | | | | | | |
| **HA Component Name** | **Unique Peptides** | **Retention Time (min)** | **Calc.M** | **Charge States** | **Batch 1** | **Batch 2** | | **Batch 3** | | **Batch 4** | **Batch 5** | | **Batch 6** | | **Batch 7** | | **Batch 8** | **Batch 9** | **Batch 10** |
| HA2 | AFDGITNMVNSVIEK | 17.1 | 1636.81 | 2,3 | 21.9 | 20.3 | | 21.2 | | 24.1 | 23.5 | | 24.3 | | 25.8 | | 19.6 | 17.5 | 20.0 |
| HA5 | AIDGVTNMVNSIIDK | 16.5 | 1588.81 | 2,3 | 26.0 | 22.1 | | 21.2 | | 26.8 | 27.9 | | 27.2 | | 27.4 | | 28.7 | 26.8 | 29.7 |
| HA7 | STQSAIDQITGMVNR | 14.4 | 1619.79 | 2,3 | 26.2 | 28.2 | | 29.6 | | 23.4 | 22.9 | | 22.5 | | 21.4 | | 22.9 | 26.8 | 24.4 |
| HA10 | STQAAIDQITGMVNR | 14.0 | 1603.8 | 2,3 | 25.9 | 29.4 | | 28 | | 25.6 | 25.7 | | 26.1 | | 25.3 | | 28.8 | 29.0 | 25.9 |

Table S2: Ratio of SteMos vaccine HA components determined based on the relative abundance of unique peptides of each of the components by MAM. A, ratio of HA components on QC samples mixed at different ratios, B, ratios of HA components on different batches of SteMos nanoparticle vaccine.

Table S3: Relative abundances of subunit and fragment peaks of HIV Trimer subunits GP120 and GP41, detected by iMAM search (stage 2)

| **Name** | **Expected mass** | **HIV Trimer**  **5 °C for 6 weeks (%)** | **HIV Trimer**  **40 °C for 1 month (%)** | **HIV Trimer**  **40 °C for 2.5 months (%)** | **HIV Trimer**  **40 °C for 5 months (%)** |
| --- | --- | --- | --- | --- | --- |
| **GP120** | | | | | |
| gp120 | 53516 | 89.21 | 86.79 | 65.48 | 51.35 |
| gp120 2-473(N.STAEN···) | 53402 | 5.59 | 7.91 | 20.47 | 21.78 |
| gp120 3-473(S.TAEN···) | 53322 | 5.17 | 5.3 | 7.89 | 14.72 |
| gp120 1-472(···NLTHH.M) | 53385 |  |  | 6.16 | 12.15 |
| **GP41** | | | | | |
| gp41, | 17510.7 | 97.56 | 93.72 | 89.67 | 82.42 |
| gp41 1-155(···DNLTLPNS.T) | 17408.6 | 1.32 | 0.71 | 1.34 | 1.73 |
| gp41 1-154(···KDNLTLPN.S) | 17321.5 | 1.12 | 4.42 | 7.43 | 9.16 |
| gp41 3-156(V.GLGAVFLG···) | 17340.5 |  | 1.15 | 1.56 | 4.22 |
| gp41 2-156(A.VGLGAVFL···) | 17439.6 |  |  |  | 1.47 |
| gp41 1-145(···SQFQQEIN.E) | 16295.4 |  |  |  | 0.98 |


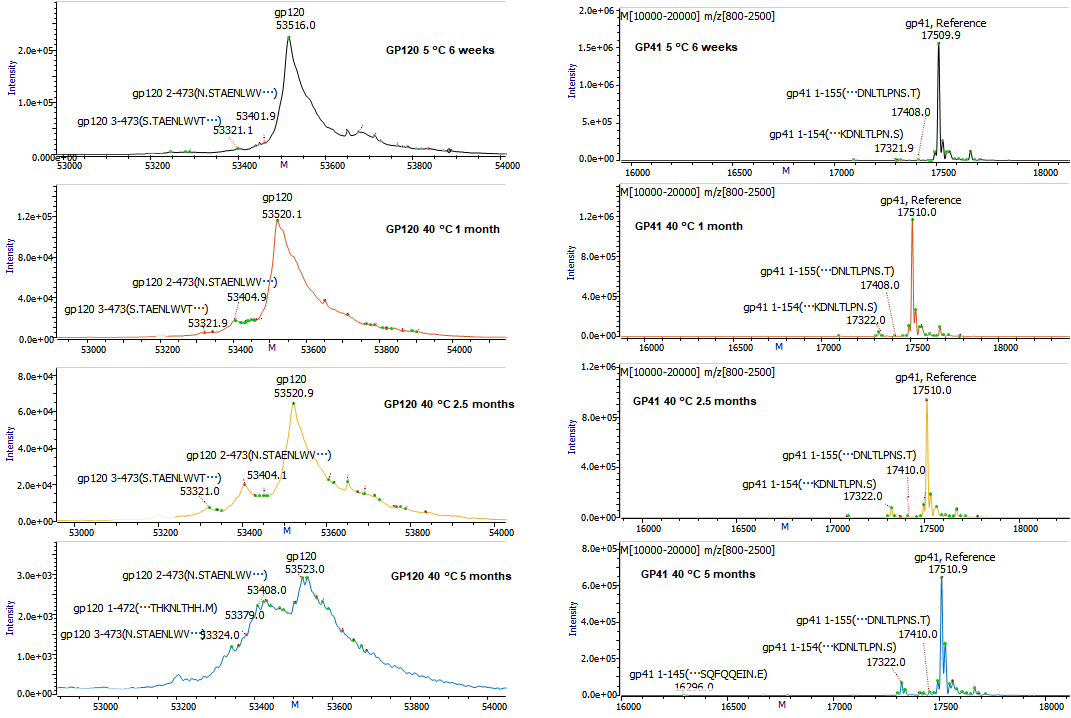


Figure S45: Deconvoluted peaks of intact subunit and fragments of GP120 and GP41 from heat stressed and control HIV Trimer subunit vaccine, detected by iMAM search using Byos software (stage 2).
